# A de novo chromosome‐level genome assembly of *Coregonus* sp. “*Balchen*”: One representative of the Swiss Alpine whitefish radiation

**DOI:** 10.1111/1755-0998.13187

**Published:** 2020-05-29

**Authors:** Rishi De‐Kayne, Stefan Zoller, Philine G. D. Feulner

**Affiliations:** ^1^ Department of Fish Ecology and Evolution Centre of Ecology, Evolution and Biogeochemistry EAWAG Swiss Federal Institute of Aquatic Science and Technology Kastanienbaum Switzerland; ^2^ Division of Aquatic Ecology and Evolution Institute of Ecology and Evolution University of Bern Bern Switzerland; ^3^ Genetic Diversity Centre (GDC) ETH Zürich Zürich Switzerland

**Keywords:** Alpine whitefish, Coregonus, genome assembly, Salmonidae, whitefish

## Abstract

Salmonids are of particular interest to evolutionary biologists due to their incredible diversity of life‐history strategies and the speed at which many salmonid species have diversified. In Switzerland alone, over 30 species of Alpine whitefish from the subfamily Coregoninae have evolved since the last glacial maximum, with species exhibiting a diverse range of morphological and behavioural phenotypes. This, combined with the whole genome duplication which occurred in the ancestor of all salmonids, makes the Alpine whitefish radiation a particularly interesting system in which to study the genetic basis of adaptation and speciation and the impacts of ploidy changes and subsequent rediploidization on genome evolution. Although well‐curated genome assemblies exist for many species within Salmonidae, genomic resources for the subfamily Coregoninae are lacking. To assemble a whitefish reference genome, we carried out PacBio sequencing from one wild‐caught *Coregonus* sp. “*Balchen*” from Lake Thun to ~90× coverage. PacBio reads were assembled independently using three different assemblers, falcon, canu and wtdbg2 and subsequently scaffolded with additional Hi‐C data. All three assemblies were highly contiguous, had strong synteny to a previously published *Coregonus* linkage map, and when mapping additional short‐read data to each of the assemblies, coverage was fairly even across most chromosome‐scale scaffolds. Here, we present the first de novo genome assembly for the Salmonid subfamily Coregoninae. The final 2.2‐Gb wtdbg2 assembly included 40 scaffolds, an N50 of 51.9 Mb and was 93.3% complete for BUSCOs. The assembly consisted of ~52% transposable elements and contained 44,525 genes.

## 1 INTRODUCTION

Members of the genus *Coregonus*, known as lake whitefish, are distributed throughout freshwater systems across Europe and North America (Bernatchez & Dodson, 1990; Douglas, Brunner, & Bernatchez, 1999; Hudson et al., 2011; Kottelat & Freyhof, 2007; Østbye, Bernatchez, Naesje, Himberg, & Hindar, 2005). In many lakes across their range, multiple whitefish species have evolved in the last 12,000 years following the melting of glaciers after the last glacial maximum (Hudson et al., 2011; Kottelat & Freyhof, 2007; Lu & Bernatchez, 1999). Today a particularly speciose clade of whitefish is found throughout pre‐alpine lakes across Switzerland, known as the Alpine whitefish radiation (Vonlanthen et al., 2012). Over 30 species are thought to make up this radiation, which was previously described as the *Coregonus lavaretus* spp. complex, and new studies continue to identify additional cryptic diversity within the radiation using genetic methods (Doenz, Bittner, Vonlanthen, Wagner, & Seehausen, 2018; Hudson, Lundsgaard‐Hansen, Lucek, Vonlanthen, & Seehausen, 2017; Hudson et al., 2011; Østbye et al., 2005). Within Switzerland, independent, monophyletic, radiations of up to six species have evolved rapidly following the last glacial maximum (Doenz et al., 2018; Hudson et al., 2011). Sympatric whitefish species in these lakes are differentiated in many phenotypic traits including body size and gill‐raker number (linked to their feeding ecology) as well as spawning depth and season (Doenz et al., 2018; Hudson et al., 2017; Kottelat & Freyhof, 2007). Repeated phenotypic differentiation has evolved independently across different lake systems, resulting in allopatric species exhibiting analogous life history strategies; for example, large, shallow spawning, benthic macro‐invertebrate eaters *C*. sp. "*Bodenbalchen*" sp. nov., *C*. sp. “*Balchen*” and *C. duplex* are present in lakes Luzern (Reuss system), Thun/Brienz (Aare system) and Walen/Zurich (Limmat system), respectively. Likewise, in the same lakes, *C. zugensis*, *C. albellus* and *C. heglingus*, small bodied pelagic zooplanktivores with high numbers of gill rakers, have also evolved, alongside up to four other sympatric species. This rapid and repeated evolution of multiple whitefish phenotypes and life history strategies has made the Alpine whitefish a particularly interesting system in which to study the genomic basis of adaptation and speciation. The recent use of genomic data gained from reduced representation libraries has demonstrated the power of genomic approaches for species designation amongst closely related sympatric species (Feulner & Seehausen, 2019). Further, it was demonstrated that genetic differentiation across the genome is widespread when comparing sympatric species from contrasting habitats (Feulner & Seehausen, 2019). However, the low density and uncertainty of positioning of markers along the genome currently still limits a true genome‐wide view of adaptation and speciation within these radiations.

The Salmonidae are a focal family in which to study genome evolution, specifically the rediploidization process following whole genome duplication. As part of the family Salmonidae, Coregonids share a common ancestor with the Salmoninae and Thymallinae. Before these subfamilies split from one another, the whole lineage experienced a whole genome duplication 80–100 million years ago (Lien et al., 2016; Macqueen & Johnston, 2014; Near et al., 2012). Recent studies have determined that different Salmonid genomes were uniquely shaped by rediploidization following this whole genome duplication, referred to as the salmonid‐specific fourth vertebrate whole‐genome duplication, Ss4R (Robertson et al., 2017). It has been shown that whilst many regions of Salmonid genomes rediploidized prior to the diversification of the three subfamilies, and thus are shared across the family, each lineage also has unique patterns of rediploidization for some genomic regions leading to substantial variation in genome structure between lineages (Robertson et al., 2017). To fully understand the impact of whole genome duplication and subsequent rediploidization on genome structure in the Salmonidae, high‐quality genome assemblies for all major lineages are needed.

Although many salmonid species now have suitably well‐assembled and curated reference genomes, including Atlantic salmon (*Salmo salar*; Lien et al., 2016), rainbow trout (*Oncorhynchus mykiss*; Berthelot et al., 2014; Pearse et al., 2019), Chinook salmon (*Oncorhynchus tshawytscha*; Christensen, Leong, et al., 2018), coho salmon (*Oncorhynchus kisutch*; NCBI BioProject: PRJNA352719), Arctic charr (*Salvelinus alpinus*; Christensen, Rondeau, et al., 2018) and European grayling (*Thymallus thymallus*; Sävilammi et al., 2019; Varadharajan et al., 2018), genomic resources for the subfamily Coregoninae are largely limited. To date, the best curated genomic resources for the Coregoninae are next‐generation sequencing linkage maps, one for the North American whitefish *Coregonus clupeaformis* (Gagnaire, Normandeau, Pavey, & Bernatchez, 2013), one for the cisco *Coregonus artedi* (Blumstein et al., 2020) and one for the Alpine whitefish radiation (*Coregonus* sp. “*Albock*”; De‐Kayne & Feulner, 2018). Here we add to the genomic resources available for Coregonids by producing a chromosome‐scale genome assembly for one species of Swiss Alpine whitefish, *Coregonus* sp. “*Balchen*.” To produce the best assembly, we tested three of the best and widely used assemblers, falcon, canu and wtdbg2 with ~90× PacBio data, validated each of the resulting assemblies, and selected the best assembly for annotation. The final assembly was produced using wtdbg2 and 94% of its total length was assembled into 40 scaffolds, in addition to 7,815 unassigned contigs. This assembly was shown to be made up of ~52% transposable elements (TEs) and contained 93.3% of complete BUSCOs (benchmarking universal single‐copy orthologues) and a total of 44,525 genes.

## 2 MATERIALS AND METHODS

### 2.1 Sample preparation and sequencing

DNA was extracted in multiple batches from heart and somatic muscle tissue of one wild caught (outbred) female *Coregonus* sp. “*Balchen*” from Lake Thun (in December 2016) using the MagAttract HMW DNA Kit (Qiagen). From this high‐molecular‐weight DNA, 45 μg was used to prepare nine libraries for sequencing on the single‐molecule real‐time sequencing (SMRT) platform from Pacific Biosciences (Sequel with 2.0 chemistry) using 60 SMRT cells to generate 240 Gb of sequence data (Next Generation Sequencing Platform, University of Bern). In addition, one Illumina TruSeq library was sequenced (paired‐end reads of 150 bp; average fragment size for Illumina library preparation 582 bp) on the Illumina HiSeq 3000 platform (Next Generation Sequencing Platform, University of Bern) which generated 87 Gb of data. These Illumina reads were evaluated for quality using fastqc (Andrews, 2010) before being used for assembly polishing.

### 2.2 Estimation of genome size

To estimate genome size for the focal species *C*. sp. “*Balchen*”, we used jellyfish version 1.1.11 (Marçais & Kingsford, 2011) to produce frequency distributions of 17‐, 21‐, 25‐ and 30‐mers for all Hi‐Seq reads. genomescope version 1 (Vurture et al., 2017) was then used to estimate genome size from these histograms.

### 2.3 Genome assembly and polishing

**Figure 1:**
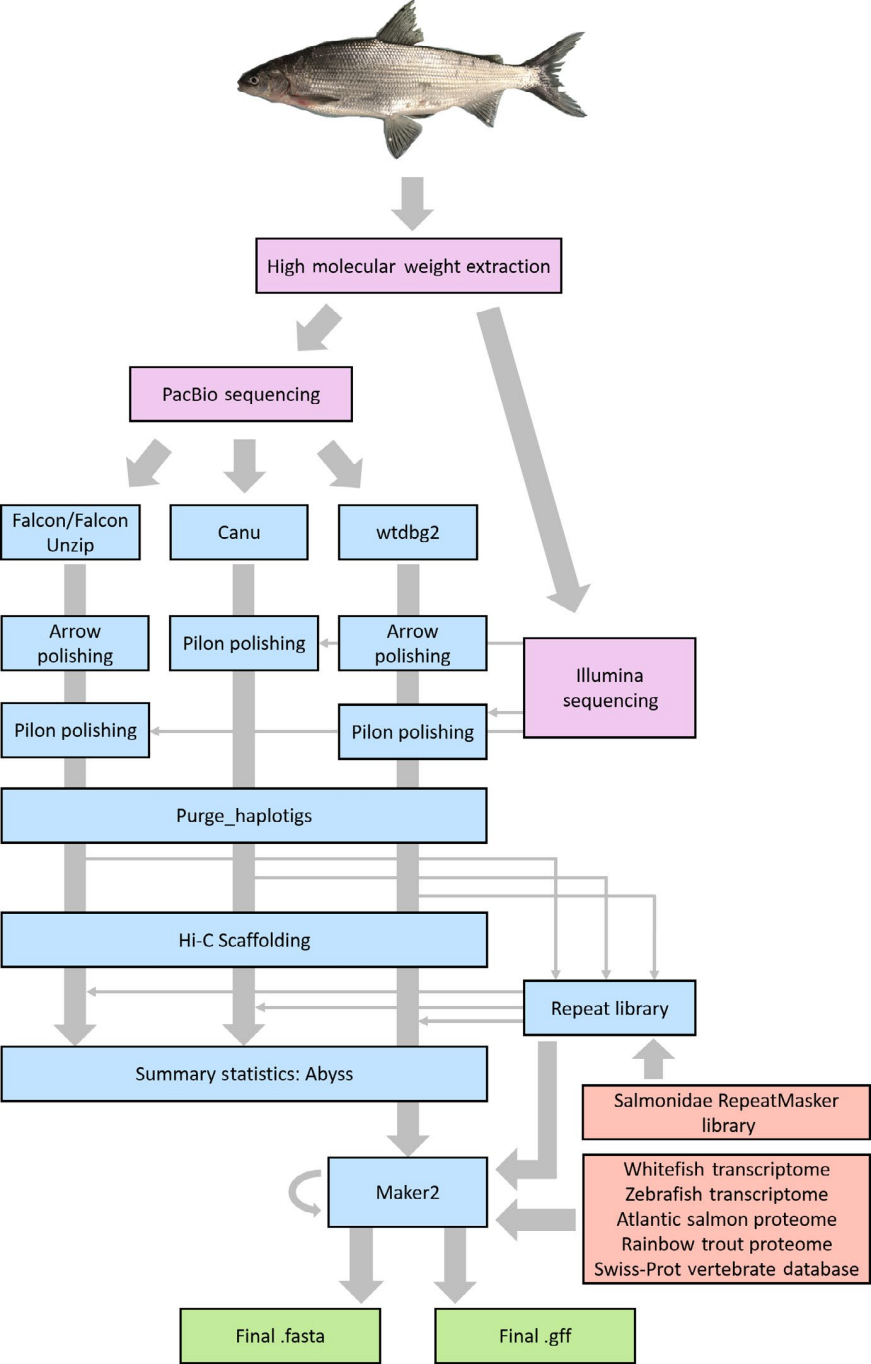
Workflow outlining the different steps and tools used to assemble the whitefish genome (coloured in blue). New input produced for this study is coloured in purple and previously published resources used for repeat masking and annotation in orange. Final outputs are shown in green [Colour figure can be viewed at wileyonlinelibrary.com]

Raw PacBio data were assembled independently using three different assemblers (Figure 1), falcon/falcon unzip version 1.9.1 (Chin et al., 2016), canu version 1.6 (Koren et al., 2017) and wtdbg2 version 2.2 (Ruan & Li, 2020), which have each demonstrated their ability to produce highly contiguous assemblies. In all three cases assembly was carried out using only PacBio data. Read polishing was carried out using both the original raw PacBio reads (arrow; SMRT link version 5.0.1; https://github.com/PacificBiosciences/GenomicConsensus) and low‐error‐rate, short‐read, Illumina data (pilon version 1.22; Walker et al., 2014). After each round of polishing, busco version 3.0.2 (Simão, Waterhouse, Ioannidis, Kriventseva, & Zdobnov, 2015) was run against the core gene set from ray‐finned fishes (actinopterygii_odb9) to evaluate the improvement of the assembly. If the number of complete BUSCOs did not improve after running the arrow algorithm, then only pilon was used. A second round of pilon polishing was also used for the wtdbg2 assembly where an additional BUSCO improvement was observed (whereas no improvement was observed after a second round of pilon polishing of the canu assembly). All assembly parameters can be found at https://github.com/RishiDeKayne/WhitefishReferenceGenome.

#### 2.3.1 falcon/falcon unzip

Genome assembly was carried out by DNAnexus utilizing the damasker suite (https://github.com/thegenemyers/DAMASKER) and the falcon (version 1.9.1) pipeline (Pacific Biosciences; Chin et al., 2016). First the REPmask and TANmask modules of the damasker suite were used on the raw PacBio reads and the resulting output was used as input for the falcon 1.9.1 pipeline. For the first two steps of the falcon pipeline, error‐correction and read overlap identification, a length cut‐off of 5,000 bp was used. This assembly was then phased and polished using falcon‐unzip (Chin et al., 2016) and Pacific Biosciences’ Arrow algorithm (https://github.com/PacificBiosciences/GenomicConsensus). The final step involved polishing these contigs using ~33× Illumina reads in the pilon program version 1.22 (Walker et al., 2014). This resulted in primary contigs, thought to represent the haploid whitefish genome, and haplotig contigs, thought to represent alternative alleles at heterozygous sites in the subject fish. For downstream processing of the falcon assembly, this primary contigs assembly was combined with the reads identified as haplotigs by falcon unzip. This allowed us to find misidentified primary contigs (which may rather represent haplotigs or mitochondrial DNA) as well as misidentified haplotigs (which could in fact be low‐coverage contigs or repetitive/duplicated regions).

#### 2.3.2 canu

Assembly following the canu (version 1.6) pipeline (Koren et al., 2017) was carried out using the same raw PacBio data. canu assembly includes three main steps, error correction followed by read trimming and, finally, assembly. canu read correction was carried out using the default settings regarding minimum read length (1,000 bp) and minimum read overlap (500 bp) whilst specifying a genome size of 4 Gb (aimed at minimizing the potential collapsing of sequence‐similar homeologous regions of the genome). The same parameters were used for the trimming step, but for the assembly step minimum read length was extended to 1,200 bp and minimum read overlap to 600 bp. Similar to the falcon/falcon unzip assembly, the final step involved polishing contigs using ~33× Illumina reads in the pilon program version 1.22 (one round; Walker et al., 2014).

#### 2.3.3 wtdbg2

finally, an assembly was carried out with the least computationally intensive of the three programs, wtdbg2 (version 2.2; Ruan & Li, 2020). wtdbg2 involves two steps, the first of which assembles long reads and the second derives a consensus sequence. For long read assembly, kmer psize was set to 21 (‐p 21), and 1/3 kmers were subsampled (using ‐S 3), the maximum length variation of two aligned fragments was set to 0.05 (‐s 0.05) and the minimum length of alignment was 5,000 bp (‐L 5,000). After the consensus was derived one round of polishing was carried out using arrow (version 5.0.1) followed by two rounds of polishing with pilon version 1.22 (Walker et al., 2014).

### 2.4 Haplotig purging

Following each assembly, we used purge_haplotigs version 1 (Roach, Schmidt, & Borneman, 2018) to identify contigs that were more likely to represent alternative alleles (from heterozygous regions of the genome) or mitochondrial DNA rather than the haploid nuclear genome. In each case, all raw PacBio data were mapped against the assembly and a read‐depth histogram was produced. A low, mid‐ and high value of coverage was then selected from this histogram to flag suspect haplotigs and regions with exceptionally high coverage, which should minimize the likelihood of removing sequence similar homeologous regions (all thresholds and histograms can be found in Table S1). Suspect haplotigs were then mapped to the rest of the assembly to identify their allelic partner before the contigs with good matches were reassigned as haplotigs. To assess the gene‐level completeness of each assembly after running purge_haplotigs each assembly was again compared against the core gene set from ray‐finned fishes (actinopterygii_odb9) using busco version 3.0.2 (Simão et al., 2015).

### 2.5 Genome scaffolding

Hi‐C scaffolding of the purged assemblies, including tissue processing, library preparation and sequencing, was carried out by Phase Genomics. Chromatin conformation capture data was generated using a Phase Genomics Proximo Hi‐C Animal Kit, which is a commercially available version of the Hi‐C protocol (Lieberman‐Aiden et al., 2009). Following the manufacturer's instructions for the kit, intact cells from the same whitefish female were crosslinked using a formaldehyde solution, digested using the *Sau*3AI restriction enzyme, and proximity‐ligated with biotinylated nucleotides to create chimeric molecules composed of fragments from different regions of the genome that were physically proximal in vivo, but not necessarily genomically proximal. Continuing with the manufacturer's protocol, molecules were pulled down with streptavidin beads and processed into an Illumina‐compatible sequencing library. Sequencing was performed on an Illumina HiSeq 4000, generating a total of 249,544,461 100‐bp read pairs.

Reads were aligned independently to each of the three draft assemblies (canu, falcon and wtdbg2). Briefly, reads were aligned using bwa‐mem version 0.7.17 (Li & Durbin, 2010) with the −5SP and ‐t 8 options specified, and all other options default. samblaster (Faust & Hall, 2014) was used to flag PCR duplicates, which were later excluded from analysis. Alignments were then filtered with samtools version 1.9 (Li et al., 2009) using the ‐F 2,304 filtering flag to remove nonprimary and secondary alignments and matlock (https://github.com/phasegenomics/matlock) using default options. Putative misjoined contigs were broken using juicebox (Durand et al., 2016) based on the Hi‐C alignments; 11 breaks were introduced into the canu assembly, 42 breaks into the falcon assembly and 11 breaks into the wtdbg2 assembly.

Phase Genomics' Proximo Hi‐C genome scaffolding platform was used to create scaffolds from each draft assembly in a method similar to that described by Bickhart et al. (2017). As in the lachesis method (Burton et al., 2013), this process computes a contact frequency matrix from the aligned Hi‐C read pairs, normalized by the number of *Sau*3AI restriction sites (GATC) on each contig, and constructs scaffolds in such a way as to optimize expected contact frequency. For each of the four assemblies, ~100,000 separate Proximo runs were performed to optimize the number of scaffolds and the scaffold construction in order to make the scaffolds as concordant with the observed Hi‐C data as possible. Finally, each set of scaffolds was polished an additional time using juicebox (Durand et al., 2016).

The differential log‐likelihood of each set of scaffolds was calculated and examined in the same manner demonstrated by lachesis. A threshold of 100 was used to identify contigs scaffolded in a position and orientation in which the log‐likelihood (base e) of the chosen orientation was more than 100 times greater than the alternative, a method shown by Burton et al. (2013) to be effective for identifying contigs that are well ordered and orientated in their region of a scaffold. Following scaffolding each of the assemblies, busco version 3.0.2 (Simão et al., 2015) was run again on each complete assembly as well as the 40 scaffolds (denoted by WFSs, CFs and FSs for the wtdbg2, canu and falcon assemblies, respectively).

### 2.6 Validation of whitefish assemblies

#### 2.6.1 Illumina short read mapping

To assess the qualitative differences between the three scaffolded assemblies we used two independent data sets, the Illumina short reads and a previously published *Coregonus* sp. “*Albock*” linkage map (see next section). Mapping the Illumina data helped to assess the composition of each of the scaffolds. The Illumina data, collected from the same individual from which the genome was sequenced, was mapped back to each of the reference assemblies. In this way we assessed the consistency of coverage across the assembly and identify potentially duplicated regions of the whitefish genome which may have been collapsed into one sequence during the assembly process. Illumina reads were mapped to each assembly using bwa‐mem version 0.7.17 (Li & Durbin, 2010; with default parameters). A summary of this mapping was produced using samtools (Li et al., 2009; samtools flagstat). Coverage was then calculated in 30‐kb windows using bedtools version 2.27.1 (Quinlan & Hall, 2010) and a custom perl script, cov.per.window.pl. Coverage statistics were then calculated in r (R Core Team, 2017).

#### 2.6.2 Linkage map synteny

In addition, we were able to assess the reliability of scaffolding by investigating the synteny between the 40 scaffolds from each assembly and the *C*. sp. “*Albock*” linkage groups (De‐Kayne & Feulner, 2018). RAD loci (90 bp containing a marker) with a known position in the linkage map were mapped to the 40 scaffolds constituting each of the three assemblies using bwa‐mem version 0.7.17 (Li & Durbin, 2010; with default parameters). Synteny plots were then visualized using the *circlize* package in r (Gu, Gu, Eils, Schlesner, & Brors, 2014; R Core Team, 2017).

### 2.7 Repeat masking and genome annotation

To characterize the repeat landscape of the whitefish genome we first produced a repeat library using repeatmodeler version 1.0.11 (Smit & Hubley, 2008) for each of the haplotig‐purged assemblies. These libraries were then combined with a Salmonidae repeat library (from repeatmasker repeat database; queryRepeatDatabase.pl ‐species Salmonidae) to produce one concatenated reference library. Each of the scaffolded assemblies was then repeat masked using this concatenated library with repeatmasker version 4.0.7 (Smit, Hubley, & Green, 2015). An interspersed repeat landscape was then produced for the best assembly, from wtdbg2, using the repeatmasker scripts calcDivergenceFromAlign.pl and createRepeatLandscape.pl.

Annotation of the wtdbg2 assembled genome was carried out using a three‐pass iterative approach with maker2 version 2.31 (Holt & Yandell, 2011). First, an initial gene model was made using our repeat library (described above), protein evidence from *Salmo salar* (UPID: UP000087266) and *Oncorhynchus mykiss* (UPID: UP000193380) proteomes from Uniprot and the Swissprot vertebrate database (uniprot_sprot_vertebrates), a recently published whitefish transcriptome (Carruthers et al., 2018) and alternative transcriptome evidence from a *Danio rerio* transcriptome (TSA: GDQQ01000001:GDQQ01083602). Next, this gene model was used to produce hidden Markov models with snap (Korf, 2004) and augustus version 3.2.1 (Stanke, Diekhans, Baertsch, & Haussler, 2008). A second pass of maker2 was then carried out using these ab initio gene prediction models and the models were optimized before a final third maker2 pass was carried out. These final maker2 gene models were filtered to remove spurious genes with Annotation Edit Distance (AED) scores < 0.6 (in accordance with Campbell, Holt, Moore, & Yandell, 2014). Finally, functional annotation of this gene set was carried out using pannzer2 (Törönen, Medlar, & Holm, 2018) and the accuracy of the annotation was determined using busco version 3.0.2 (Simão et al., 2015; with the core gene set from ray‐finned fishes actinopterygii_odb9) on the final gene set. To further evaluate our gene set we used orthofinder version 2.3.11 (Emms & Kelly, 2015, 2019) to construct orthologous gene sets. This analysis included protein sequences from whitefish (42,695 genes annotated with an AED < 0.6 and positioned on the 40 wtdbg2 whitefish scaffolds) and three other salmonids (*Hucho hucho* [ASM331708v1; GCA_003317085.1; submitted by University of Aberdeen in July 2018], *Salmo salar* [ICSASG_v2; GCA_000233375.4; submitted by International Cooperation to Sequence the Atlantic Salmon Genome in June 2015], *Salmo trutta* [fSalTru1.1; GCA_901001165.1; submitted by SC in June 2019]) and the outgroup (which did not go through the Ss4R whole genome duplication) *Esox lucius* (Eluc_V3; GCA_000721915.3; submitted by Ben F. Koop and Jong S. Leong in January 2017). All protein files were downloaded from ENSMBL (https://www.ensembl.org/index.html; 21.Feb 2020).

### 2.8 Identification of homeologous regions in the whitefish genome

Following the whole genome duplication that occurred in an ancestral salmonid it is possible to determine which whitefish scaffolds (WFSs; many of which correspond to chromosomes) are homeologues of one another by identifying pairs of scaffolds that are sequence similar. After hard‐masking the WFSs resulting from the wtdbg2 assembly and scaffolding we aligned each WFS to all other WFSs in symap version 5.0 (Soderlund, Bomhoff, & Nelson, 2011; Soderlund, Nelson, Shoemaker, & Paterson, 2006), using default parameters. For each of the 55 links between homeologous WFS blocks identified in symap, lastz version 1.02 (Harris 2007) was run in both directions (using the parameters: ‐‐gfextend ‐‐nochain ‐‐nogapped ‐‐matchcount = 100; similarly to Lien et al., 2016) to align these regions to one another and subsequently determine sequence similarity between the two. Following lastz alignment, matches were filtered to remove those with sequence similarity < 75% (in keeping with Lien et al., 2016) and/or smaller than 1,000 bp, and sequence similarity was then averaged across alignments within each block.

### 2.9 Ancestral chromosome identification

We also aimed to determine the single (nonduplicated) ancestral chromosome that each homeologous pair of scaffolds corresponds to, and subsequently determine the level to which chromosomal rearrangements may have taken place in whitefish. Each wtdbg2 wfS was mapped to the northern pike (*Esox lucius*) genome (GCF_004634155.1) using symap version 5.0 (Soderlund et al., 2006, 2011) following the ancestral chromosome identification convention used by Sutherland et al. (2016) and Blumstein et al. (2020). The identified syntenic links between WFSs and pike chromosomes then allowed us to determine which ancestral pike chromosome (PK; pre‐whole genome duplication) corresponds to each homeologous pair of WFSs (identified above; as the result of whole genome duplication) or to a single WFS, evidential of one copy of a pair having been lost.

## 3 RESULTS

### 3.1 Estimation of genome size

**Figure 2:**
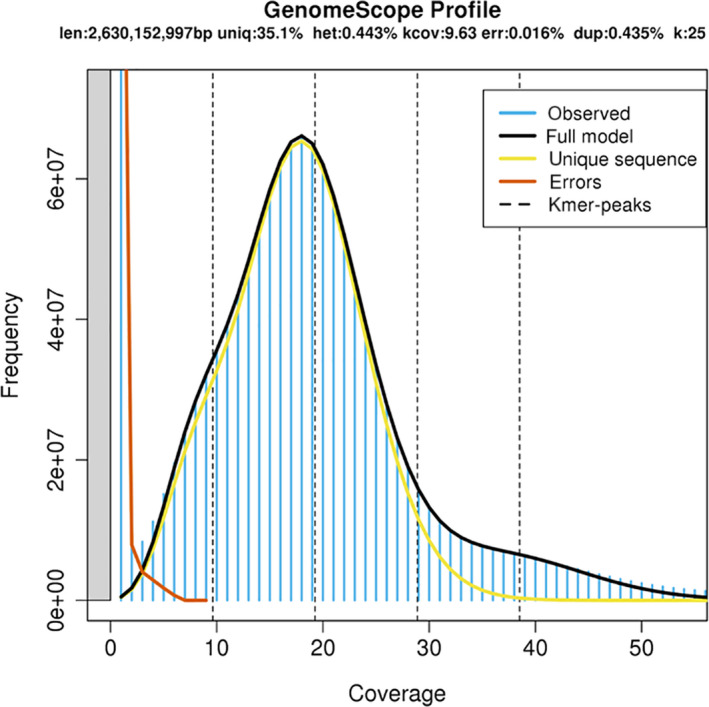
genomescope profile established based on short read data, which estimates the genome size of *Coregonus* sp. “*Balchen*” to be 2.6 Mb [Colour figure can be viewed at wileyonlinelibrary.com]

Reports of *Coregonus* genome sizes vary widely with estimates ranging from 2.4 Gb (Hardie & Hebert, 2003) to 3.5 Gb (Lockwood, Seavey, Dillinger, & Bickham, 1991). Using the best model (*k* = 25), as determined using the ‘Model Fit’ output from genomescope, we estimated *C*. sp. “*Balchen*” to have a genome size of 2.63 Gb (Figure 2). Based on this estimate of genome size the PacBio sequencing used equates to ~91× coverage (and the Illumina HiSeq to ~33×).

### 3.2 Genome assembly and polishing

After the first step of the falcon pipeline, prior to phasing and polishing with falcon unzip the assembly contained 52,448 primary contigs covering 2.78 Gb with an N50 contig length of 204 kb. After the falcon unzip step the primary contigs assembly was made up of 19,553 contigs covering 2.41 Gb and with an N50 of 280 kb (Table 1). For downstream analysis this primary contigs assembly was merged with the haplotigs assembly, as described above. This merged assembly was 4.11 Gb long, contained 60,605 contigs, had an N50 of 136 kb and included 89.5% of complete BUSCOs (Table 1; Table S1). The canu assembly was substantially larger than the primary reads from the falcon/falcon unzip assembly and covered 3.28 Gb across 52,023 contigs, with an N50 of 131 kb and including 88.7% BUSCOs (Table 1; Table S1). The wtdbg2 assembly was the shortest of the three with a total length of 2.38 Gb and also had the fewest contigs (28,224; Table 1). However, it had the highest N50 of 424 kb and contained the highest percentage of complete BUSCOs, 93.4% (Table 1; Table S1).

**Table 1:** Summary statistics at the contig (prehaplotig purging) and scaffold stage (each scaffolded assembly contains 40 scaffolds and a number of unscaffolded contigs) for the falcon, canu and wtdbg2 assemblies

	falcon (primary contigs)	canu	wtdbg2
contig statistics			
Number of contigs	60,605 (19,553)	52,023	28,224
Contig N50 (bp)	136,418 (279,657)	130,955	424,474
Longest contig (bp)	6,516,619 (6,516,619)	5,278,180	5,201,837
Total contig length (Gb)	4.11 (2.41)	3.28	2.38
Scaffolded assembly statistics			
Number of scaffolds	40	40	40
Number of unscaffolded contigs	3,705	3,513	7,815
Combined N50 (bp)	62,840,000	59,340,000	51,930,000
Longest scaffold (bp)	111,300,000	104,000,000	93,420,000
Total combined length (Gb)	2.47	2.46	2.20
Scaffolded assembly BUSCOs/40 scaffolds BUSCOs			
Complete	4,209 (91.8%)/4,195 (91.5%)	4,299 (93.7%)/4,297 (93.7%)	4,274 (93.3%)/4,263 (93%)
Single	2,713 (59.2%)/2,732 (59.6%)	2,578 (56.2%)/2,583 (56.3%)	2,551 (55.7%)/2,551 (55.7%)
Duplicated	1,496 (32.6%)/1,463 (31.9%)	1,721 (37.5%)/1,714 (37.4%)	1,723 (37.6%)/1,712 (37.3%)
Fragmented	89 (1.9%)/77 (1.7%)	78 (1.7%)/78 (1.7%)	95 (2.1%)/83 (1.8%)
Missing	286 (6.3%)/312 (6.8%)	207 (4.6%)/209 (4.6%)	215 (4.6%)/238 (5.2%)

### 3.3 Haplotig purging

After haplotig purging the differences between the three assemblies was reduced dramatically, with the range of contigs now from 16,440 to 22,627 (for wtdbg2 and canu, respectively; Table S1). The N50 of all three assemblies also increased, particularly in the falcon and canu assemblies, from 136 and 131 kb to 281 and 258 kb each. The N50 of the wtdbg2 assembly also increased, although less significantly, from 424 to 491 kb (Table S1). The number of complete BUSCOs went up in both the falcon and the canu assemblies after haplotig purging (by 1.3% and 4.4%), but dropped slightly (by 0.3%) in the wtdbg2 assembly (Table S1). The high completeness percentage of BUSCOs for each of the assemblies prior to scaffolding suggests that we have succeeded in capturing a large proportion of the whitefish genome sequence during the assembly process.

### 3.4 Genome scaffolding

**Figure 3:**
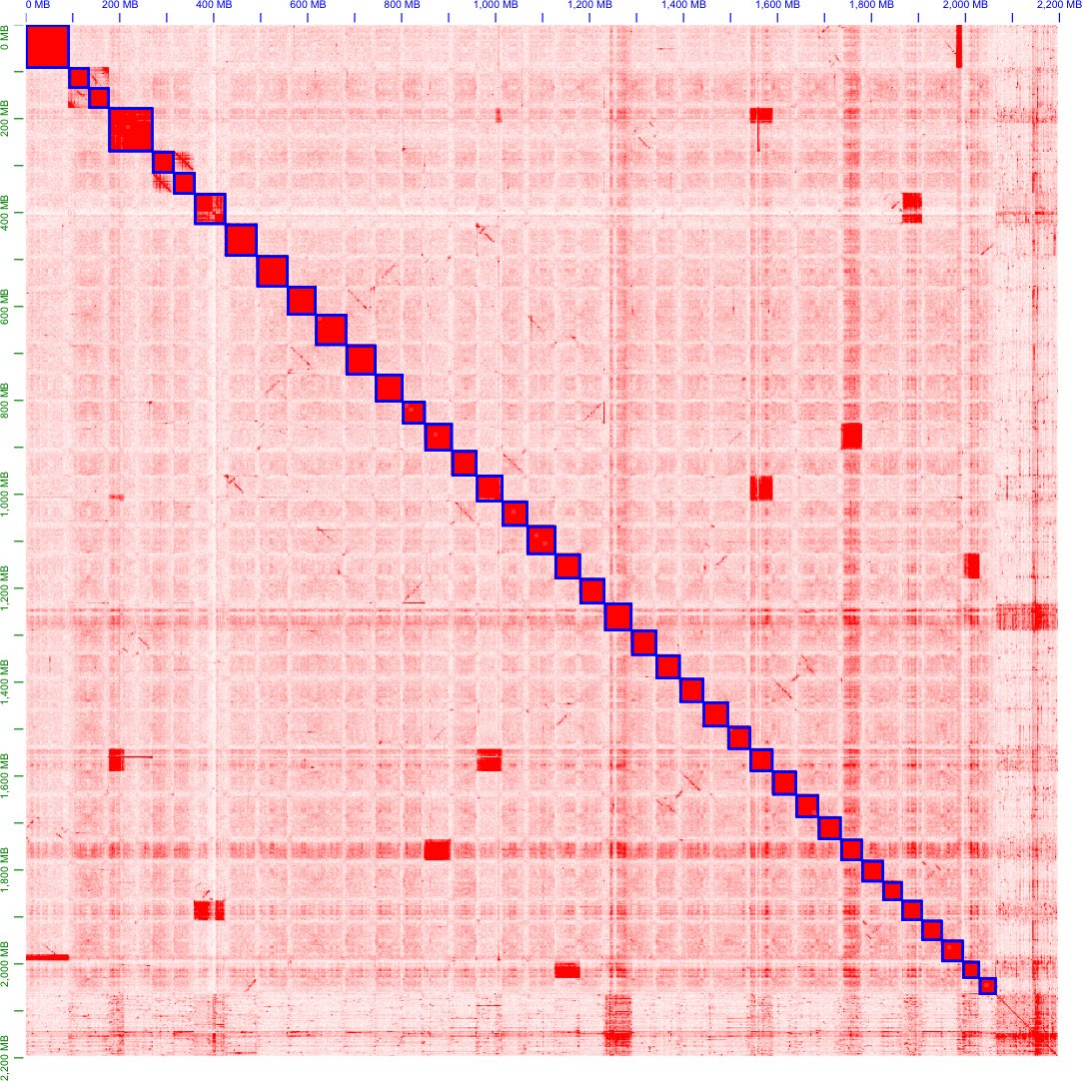
*Coregonus* sp. “*Balchen*” contig contact map from Hi‐C scaffolding of the wtdbg2 assembly. The intensity of red represents the relative contact density between contigs. The highest contact density is found within whitefish scaffolds (WFSs), which are outlined in blue [Colour figure can be viewed at wileyonlinelibrary.com]

Hi‐C scaffolding of contigs into scaffolds resulted in a set of 40 scaffolds, many of which were chromosome‐scale, for each of the three assemblies, containing 2.38 Gb (96% of all sequence; falcon), 2.41 Gb (98% of all sequence; canu) and 2.07 Gb (94% of all sequence; wtdbg2). The differential log‐likelihood calculation, which was used identify the length of confidently ordered and orientated scaffolds, resulted in 1.08 Gb (45.41%) for the falcon scaffolds (FSs), 1.21 Gb (50.1%) for the canu scaffolds (CSs) and 1.74 Gb (84.1%) for the wtdbg2 scaffolds (WFSs), meeting this criterion. These results are in agreement with the patterns observable in the final scaffold heatmaps for each assembly, in which the patterns observable for the wtdbg2 scaffolds are more in alignment with a priori expectations about Hi‐C linkage density patterns (Figure 3; contact plots for the falcon and canu assemblies are displayed in Figures S1 and S4; van Berkum et al., 2010), yielding qualitative confirmation of the quantitative scaffold quality assessment. The percentage of complete BUSCOs went up for each assembly following Hi‐C scaffolding, with the falcon, canu and wtdbg2 assemblies now having 91.8%, 93.7% and 93.3% (Table 1). When considering only scaffolds, the canu assembly retained the highest complete percentage of BUSCOs at 93.7% with the falcon and wtdbg2 assemblies dropping only slightly to 91.5% and 93.0% each. Based on having the highest length of confidently scaffolded contigs and the high number of complete BUSCOs, the Hi‐C scaffolded wtdbg2 assembly was selected as the best of the three and was uploaded to the European Nucleotide Archive (Accession no.: GCA_902810595.1). The falcon and canu assemblies are available on Dryad (https://doi.org/10.5061/dryad.xd2547ddf).

### 3.5 Validation of whitefish assemblies

#### 3.5.1 Illumina short read mapping

**Figure 4:**
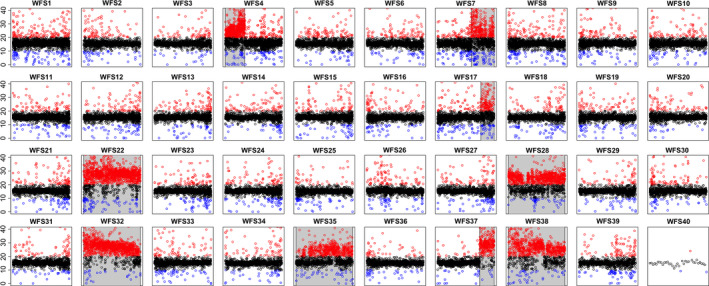
For each of the whitefish scaffolds (WFSs) coverage of Illumina data mapped to the wtdbg2 assembly is plotted in 30‐kb windows. Most windows show an average coverage of around 17× (black points). Windows with coverage > 20× and < 10× are coloured in red and blue, respectively. Putative collapsed duplicate regions are highlighted in grey [Colour figure can be viewed at wileyonlinelibrary.com]

Summaries of the mapping of Illumina reads to each of the assemblies can be seen in Table S2. The wtdbg2 assembly had the highest number of mappings over mapping quality (MAPQ) 30 (71.6%) with the canu and falcon assemblies having slightly lower proportions of high‐quality mappings (69.5% and 60.7% each). When considering the proportion of read mates mapped to a different scaffold, however, the canu assembly looks the best of the three with only 1.4% of mates mapped to a different scaffold with an MAPQ> 5, compared to 2% for falcon and 2.4% for wtdbg2. as a result of our coverage analysis the highest mean coverage (in 30‐kb windows) was 17.3× observed in the wtdbg2 assembly. The falcon and canu assemblies had lower mean coverages of 15.0× and 15.9×, respectively. Plots of coverage across the 40 wtdbg2 wfSs are shown in Figure 4 and the equivalent plots for the falcon and canu assemblies in Figures S2 and S5. Based on these coverage plots we identified regions which are likely to represent collapsed duplicated regions, spread across each genome assembly (Tables S3 and S4). As expected in these regions, coverage was approximately double that of the rest of the assembly. In other salmonid genome assemblies, which have successfully resolved each copy of a duplicated region, these duplicated regions typically span whole chromosome arms or even chromosomes. Similarly, in the whitefish assemblies we identify collapsed blocks which encompass whole scaffolds or parts of scaffolds. In the wtdbg2 assembly some WFSs (WFS4, 7, 14 and 37) probably represent collapsed regions which span chromosome arms, and in other WFSs (WFS22, 28, 32, 36 and 38) the whole chromosome appears collapsed (a BED file containing the locations of these wtdbg2 collapsed duplicates is included in Table S3). We estimate that in total 309 Mb of the wtdbg2 assembly (representing 14% of the assembly) is collapsed compared to 413 and 517 Mb in the canu and falcon assemblies, respectively (representing 17% and 21% of each assembly; Table S4).

#### 3.5.2 Linkage map synteny

**Figure 5:**
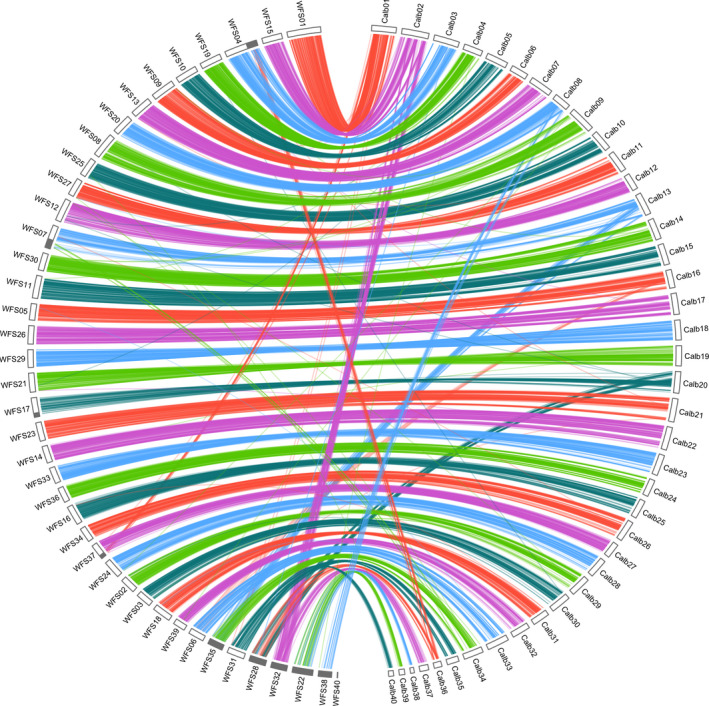
Circos plot comparing the structure of the *C*. sp. “*Albock*” linkage map (right; De‐Kayne & Feulner, 2018) and the 40 whitefish scaffolds (WFSs) of the wtdbg2 *c.* sp. “*Balchen*” assembly (left). Lines indicate mapping locations of RAD loci from the linkage map in the genome assembly. Most mappings suggest a good match between linkage map and genome assembly (high synteny between linkage groups and WFSs) and only few lines map discordantly. Genome assembly regions which represent collapsed duplicate regions are identified in grey around the left perimeter [Colour figure can be viewed at wileyonlinelibrary.com]

Out of the 5,395 markers from the *C*. sp. “*Albock*” linkage map which were mapped to each assembly, only high‐quality mappings (MAPQ> 30) were retained, resulting in a mapping position for 3,648, 4,494 and 4,744 markers in the sequence of the falcon, canu and wtdbg2 assemblies respectively (Table S2). For all three assemblies, concordance between sequence and recombination position across the majority of markers was very high, suggesting a high synteny between linkage groups and scaffolds. In the wtdbg2 assembly 95% of markers (4,489/4,744) showed strong synteny between one linkage group and one WFS (38 out of the 40 linkage groups; Figure 5; equivalent to Figures S3 and S6 for the falcon and canu assemblies, respectively). Only two scaffolds (WFS38 and 40) could not be matched to any linkage group. We also identified a series of substantial deviations from the broader pattern, where a number of markers from a linkage group also mapped to a second, alternative scaffold. This was the case for markers from Calb01 – WFS37, Calb02 – WFS32, Calb08 – WFS38, Calb13 – WFS35, Calb16 – WFS06, Calb20 – WFS28, Calb34 – WFS07 and Calb36 – WFS04. Strikingly the mapping locations of seven of these deviations (WFS04, 07, 28, 32, 35, 37, and 38) also represent seven of the nine scaffolds we identified as collapsed duplicates showing inflated coverage (shown in grey in Figures 4, 5, and 7). Although part of WFS17 resembled a collapsed duplicate based on coverage, no significant deviations of markers mapped to this scaffold. Additionally, despite having an unusual mapping pattern with markers from Calb16 in addition to those from Calb33, WFS06 showed a consistent coverage of Illumina reads mapping. Out of all deviating markers, 83% (212/255) mapped to regions identified as collapsed duplicates. No markers from the linkage map were successfully mapped to WFS40, the smallest of the scaffolds, at only 1.1 Mb long. Additionally, in two cases markers from two linkage groups predominantly mapped to one WFS. Markers from both Calb35 and Calb40 mapped to WFS31 and from Calb38 and Calb39 to WFS22. The few deviations from the patterns of synteny between linkage groups and scaffolds could be caused by a number of interacting factors. These include the potential collapse of some scaffolds or parts of scaffolds, small mistakes in either the linkage map or the sequence assembly, and the distribution of repetitive sequence similar regions which all reduce the accuracy of mapping the short (90‐bp) RAD loci from the linkage map. However, collapsed regions probably play the most significant role in driving deviations in the observed patterns of synteny, as evidenced by the fact that the majority of deviating RAD loci map to regions that are thought to be collapsed.

### 3.6 Repeat masking and genome annotation

**Figure 6:**
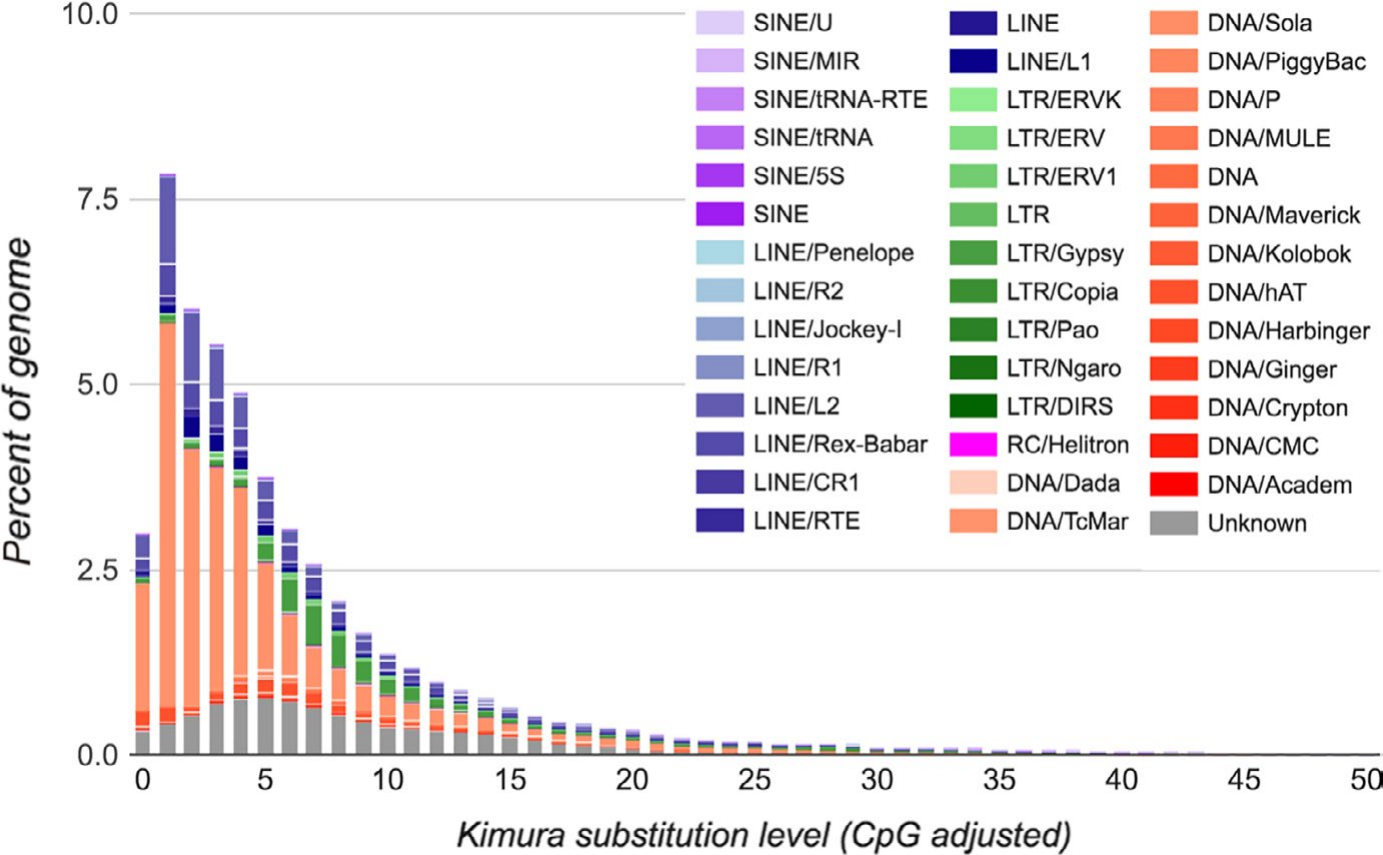
*Coregonus* sp. “*Balchen*” transposable element divergence landscape. Transposable elements within the whitefish genome have been characterized (different classes represented by distinct colours). The plot shows the relative abundance of each class and their relative age (molecular clock estimate). Note the ongoing DNA element diversification within the whitefish genome, particularly in DNA elements and LINEs [Colour figure can be viewed at wileyonlinelibrary.com]

Around 52% of each assembly was masked with the most abundant repetitive elements being DNA elements followed by Long Interspersed Nuclear Elements (LINEs) and then unclassified repeats (Table S1). DNA elements alone made up nearly a quarter of each assembly (24.65% of falcon, 23.79% of canu and 24.41% of the wtdbg2 assembly). The resulting landscape (Figure 6) identified the Class II TE superfamily Tc1‐*mariner* as the most abundant in the *Coregonus* sp. “*Balchen*” genome, making up 18% of the interspersed repeats. The most abundant Class I TEs were LINE‐2 elements, although these only made up 4.2% of the interspersed repeats. The three‐pass maker2 annotation resulted in the identification of 44,525 protein‐coding genes (42,695 on scaffolds and 1,830 on unscaffolded contigs) and included 357,479 identified exons (Table 2), with the final set of genes being 81.8% complete for BUSCOs (C: 81.8% [S: 54.8%, D: 27.0%], F: 9.3%, M: 8.9%, *n*: 4,584). Functional annotation with pannzer2 allowed the assignment of gene ontology terms to 29,046 genes. Across a total of 415,276 genes in the five species, we identified 41,042 orthogroups. This includes 7,673 species‐specific orthogroups, 725 single‐copy orthologues and 16,599 orthogroups with all five species present. Out of the 42,695 whitefish genes (on scaffolds), 38,219 could be assigned to 22,311 orthogroups. The number of whitefish genes annotated on scaffolds (42,695) is similar to the number of genes annotated in the diploid outgroup (*Esox lucius* 43,143 genes), but substantially lower than the ENSMBL annotation of any of the other three salmonids (*Hucho hucho* 91,817 genes, *Salmo salar* 121,064 genes, *Salmo trutta* 116,557 genes). This suggests that more transcriptomic work on various tissues and developmental stages is warranted to further improve the annotation of the whitefish genome. We found 4,504 orthogroups that were duplicated in whitefish (present with more than one copy) that were present in a single copy in pike (*Esox lucius*). A total of 2,746 orthogroups show duplication patterns relative to the pike that are consistent across all four salmonids. However, we also identified 3,459 orthogroups where only the other three salmonids show duplications and 474 orthogroups where only whitefish appear duplicated. This could reflect true biological differences in duplication loss in the different salmonid lineages, although technical artefacts during the assembly (collapsing of highly identical regions) or gene annotation differences probably contribute as well.

**Table 2:** Genome annotation summary statistics for final the wtdbg2 assembly following three‐pass maker2 annotation

Genes	Number	44,525
	Mean length (bp)	11,473.3
	Median length (bp)	4,850
	Min./max. (bp)	77/181,605
	Gene frequency (genes/Mb)	20.24

Exons	Number	357,479
	Mean length (bp)	196.7
	Median length (bp)	135
	Min./max. (bp)	2/17,274

### 3.7 Identification of homeologous regions in the whitefish genome

**Figure 7:**
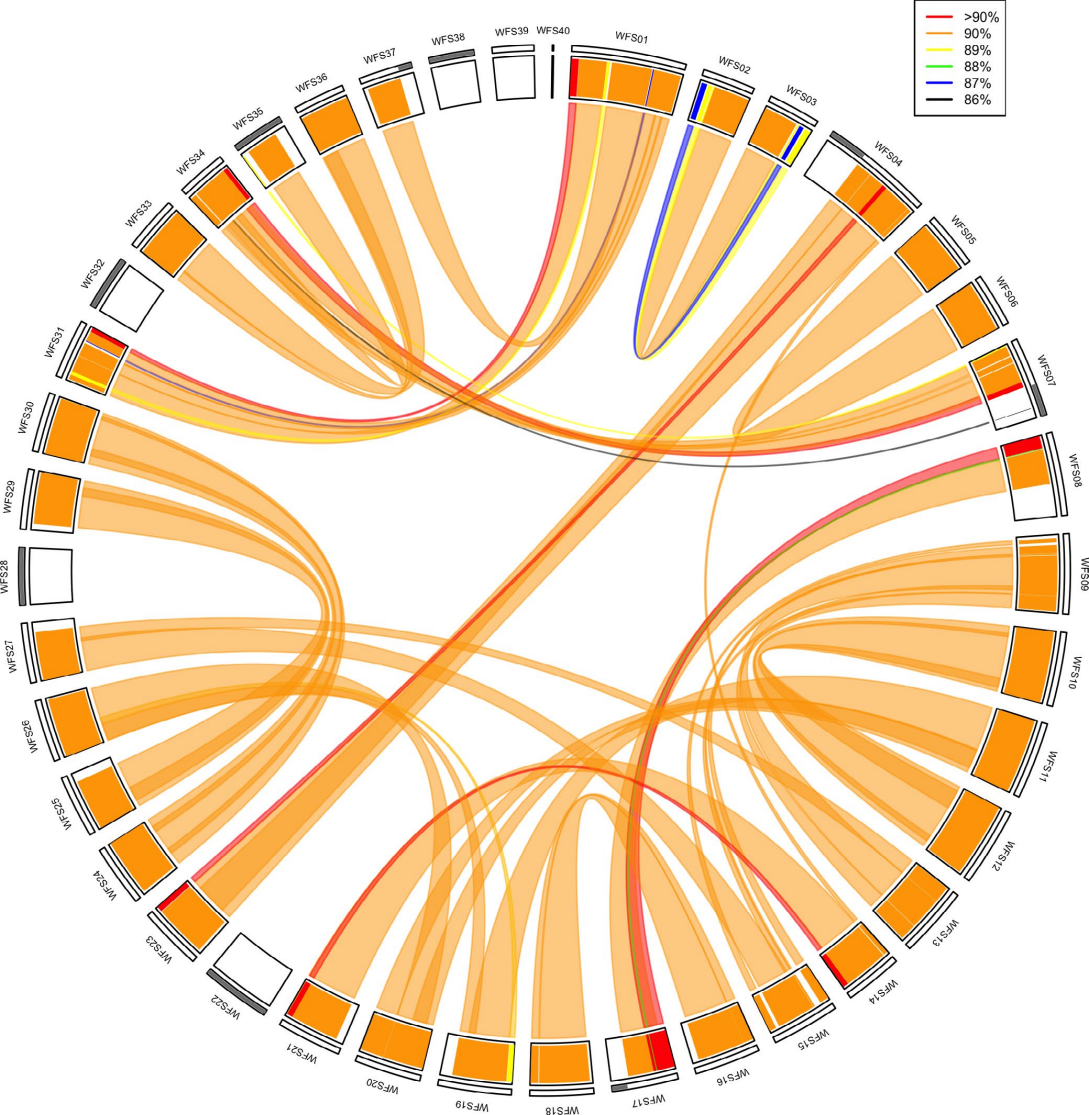
Homeologous whitefish scaffolds (WFSs) within the whitefish genome identified using symap. Links between homeologues are coloured according to their mean sequence similarity based on lastz alignments for each syntenic link in both directions. Genome assembly regions which are thought to be collapsed are identified in grey on the outermost track [Colour figure can be viewed at wileyonlinelibrary.com]

Using symap we identified 55 syntenic links between 34 of the 40 WFSs (Figure 7; Table S5). Sequence similarity calculations for each link (110 mappings, one in each direction, for each of the 55 homeologous blocks) showed that the majority of identified syntenic blocks had sequence similarity ≥ 90% (shown in orange and red in Figure 7). Slightly lower sequence similarity was observed for syntenic links between WFS02 and WFS03 and multiple smaller links between WFS01 and WFS31, WFS07 and WFS34, and WFS08 and WFS17. The WFSs for which no syntenic links were identified were WFS22, WFS28, WFS32, WFS38, WFS39, and WFS40. Of those, WFS22, WFS28, WFS32, and WFS38 have been identified as fully collapsed based on their unusual coverage patterns, and hence probably represent residual tetraploid regions or a collapse of two very sequence similar homeologues. It is likely that the shortest scaffold, WFS40, constitutes part of a chromosome, explaining why no homeologous scaffold was found.

### 3.8 Ancestral chromosome identification

As above, symap was used to identify the corresponding nonduplicated ancestral chromosome for each syntenic WFS pair. This comparison of the WFSs and the northern pike genome identified that the majority of pike chromosomes (18 out of 25) had two equivalent WFSs, and the remaining seven pike chromosomes match to only one WFS (Table 3; Figure S7). These seven pike chromosomes included PK2, PK9, PK11, PK20, and PK23 which corresponded to WFS32, WFS38, WFS35, WFS28 and WFS4. Additionally, PK22 and PK25 are both in single copy in whitefish but appear to have been rearranged into a single WFS, WFS22.

**Table 3:** Summary of synteny between whitefish scaffolds (WFSs) and northern pike (PK) chromosomes, and between WFSs

WFS	Corresponding pike—PK chromosome (secondary PK)	Homeologous WFS (secondary homeologue)
1	6 (5)	37 (31)
2	4	3
3	4	2
4	16 (23^†^)	23
5	1	6
6	1	5
7	21	34
8	10 (24)	17 (39^*^)
9	7	13
10	17	12
11	19	19
12	17	10
13	7	9
14	3	21
15	14	27
16	13	18
17	10	8
18	13	16
19	19	11
20	8	26
21	3	14
22	22^†^ (25^†^)	— (—)
23	16	4
24	12	30
25	15	29
26	8	20
27	14	15
28	20^†^	—
29	15	25
30	12	24
31	5	1
32	2^†^	—
33	18	36
34	21	7
35	11^†^	—
36	18	33
37	6	1
38	9^†^	—
39	24	8^*^
40	—	—

Note

Homology between WFSs and PK chromosomes, and between WFSs was inferred using symap. In the first column WFSs are underlined if they are thought to be collapsed. In the second column corresponding PK chromosomes are identified with a cross (†) if they were found to have homology with only one WFS. The third column indicates homeologous WFSs, which were identified when two WFSs showed synteny with the same PK chromosome (see Figure S7). An asterisk (*) highlights homeologous relationships that were not confirmed with our analysis of synteny between WFSs.

## 4 DISCUSSION

To enable future studies to investigate both the genetic basis of adaptation and speciation within Coregoninae and genome evolution following whole genome duplication across the family Salmonidae we have assembled the first whitefish reference genome. Assembling > 90× PacBio data from one female *Coregonus* sp. “*Balchen*” with three of the most commonly used assemblers resulted in three high‐quality assemblies, each with > 90% complete BUSCOs and 40 scaffolds, many of which are chromosome‐scale. Out of these three assemblies we judge the assembly produced by wtdbg2 as the best. This new draft whitefish genome is 2.2 Gb and comprises 40 scaffolds (containing 94% of nucleotides) and 7,815 unscaffolded contigs, has an N50 of 51.9 Mb and contains 93.3% complete BUSCOs. Annotation of the assembly identified 44,525 genes in total and showed that TEs make up 52% of the *C*. sp. “*Balchen*” genome.

To assemble the first reference genome from Coregoninae, we made use of three different but widely used genome assemblers, falcon/falcon unzip, canu and wtdbg2. although studies describing new assembly software often compare genome assemblies produced with the same input data and multiple different assemblers, the high cost and prohibitive computational time usually restricts these comparisons to genomes < 150 Mb, including *Arabidopsis thaliana* and *Drosophila melanogaster* (Chin et al., 2016), and those of model systems including human cell line CHM1 (Koren et al., 2017; Ruan & Li, 2020). Few studies have reported such performance comparisons with nonmodel species, despite an increasing number of de novo reference genome assembly projects for organisms with large, complex genomes. All three assemblies were subsequently polished using arrow (falcon and wtdbg2) and/or pilon (falcon, canu and wtdbg2) and scaffolded using Hi‐C technology into 40 scaffolds. At the contig stage the falcon assembly was the longest at 4.11 Gb (containing 60,605 contigs), and the wtdbg2 the shortest at 2.38 Gb (containing 28,224 contigs). Although the structure of each assembly at the contig stage varied, each of the three assemblies had high complete BUSCO percentages (Table S1). This shows that all three assemblers performed well with the input data, producing contigs which incorporate around 90% of genes known to be present in all ray‐finned fishes. After haplotig purging, the three assemblies became more similar in size and N50 value (Table S1), which suggests that the assemblers differed largely in their ability to resolve alleles. Next, Hi‐C scaffolding was used, resulting in 40 scaffolds for each assembly. For the falcon, canu and wtdbg2 assemblies, 2.38/2.47 Gb (96%), 2.41/2.46 (98%) and 2.07/2.2 Gb (94%), respectively, were assigned to the 40 scaffolds. Out of the three assemblies, more of the wtdbg2 assembly could be confidently scaffolded (84.1% meeting the criterion compared to 45.4% and 50.1% in the falcon and canu assemblies). Also, during the assembly validation process the wtdbg2 assembly appeared to be the best of the three assemblies, having the highest mean coverage across the genome and lowest proportion of the genome in potentially collapsed regions (Table S4) as well as the largest proportion of confidently mapped linkage map markers. Although the complete BUSCO scores were slightly lower than that of the canu assembly (93% in the wtdbg2 assembly compared to 93.7% in the canu assembly), the increased confidence of scaffolding in the wtdbg2 assembly, the superior mapping metrics, the lowest proportion of the genome being collapsed and highest synteny with the linkage map all led to us selecting this as the best assembly.

The scaffold N50 of the wtdbg2 *c.* sp. “*Balchen*” assembly was 51.9 Mb, which is higher than for a number of recently published salmonid genomes including Chinook salmon (1.138 Mb; Christensen, Leong, et al., 2018), Arctic charr (1.02 Mb; Christensen, Rondeau, et al., 2018) and grayling (33 Mb; Sävilammi et al., 2019). The characterization of the repeat landscape of the whitefish genome also highlighted the broad similarity in the proportion of many families of TEs between salmonid species. We identified that around 52% of the whitefish genome is repetitive, a similar proportion to that of Chinook salmon (56%; Christensen, Leong, et al., 2018), Arctic charr (56%; Christensen, Rondeau, et al., 2018) and European grayling (47%; Sävilammi et al., 2019). The relative abundances of different types of repetitive element are similar to those reported in other salmonid assemblies, including that of Atlantic salmon (Lien et al., 2016) and Chinook salmon (Christensen, Leong, et al., 2018). The relatively high abundance of Class II TE superfamily Tc1‐*mariner* and LINE‐2 elements amongst the youngest elements suggest that these families are still expanding and potentially diversifying in the whitefish genome. Conversely, the lack of new Long Terminal Repeat (LTR) elements suggests that their abundance and diversity peaked in the past and they are no longer diversifying in the genome. Annotation of the wtdbg2 assembly identified 44,525 genes, similar to those reported in the publications associated with the rainbow trout genome (46,585 by Berthelot et al., 2014; 53,383 by Pearse et al., 2019) but higher than the 37,206 genes identified by Lien et al. (2016) in the Atlantic salmon genome and the 36,216 identified by Christensen, Leong, et al. (2018) in the Chinook salmon genome.

Although whole genome duplications have punctuated the tree of life, few have occurred recently enough to allow investigations into the subsequent rediploidization process at the genomic level. Salmonids are therefore an ideal family in which to study rediploidization because the genomic signals of whole genome duplication and genomic rearrangements which followed have not yet been confounded by other genomic processes such as mutations (including small point mutations and large structural changes such as inversions and deletions; Macqueen & Johnston, 2014). One recent investigation into rediploidization within Salmonidae identified substantial genomic differences between 16 salmonid species, which evolved independently as rediploidization proceeded (Robertson et al., 2017). However, with high‐quality genomes available for an increasing number of salmonid species, the resolution with which we can identify differences that have occurred in genome structure and composition following whole genome duplication is vastly increasing. Therefore, highly contiguous reference genomes, particularly for under‐represented groups such as the salmonid subfamily Coregoninae, are invaluable to fill gaps in the genomic resources currently available. Here, we have been able to determine pairs of whitefish scaffolds that represent homeologues and their corresponding ancestral chromosome (using northern pike chromosome numbering). This will facilitate future comparisons across the salmonid family (similar to those by Blumstein et al., 2020) to assess the independent rediploidization process in different salmonid lineages. We also identified a number of whitefish scaffolds for which no homeologue was present. By combining synteny data with our coverage‐based validation of the new *C*. sp. “*Balchen*” assembly we showed that some of these regions were due to the collapsing of highly sequence‐similar regions (e.g., WFS22 and WFS28). In other instances, we identified potential genomic rearrangements as the driver of this pattern—for example, pike PK24 showed homology with WFS39 but also a part (potentially a chromosome arm) of WFS08 (Table 3; Figure S7). More complex patterns were also identified such as the merging of two different PK chromosomes (22 and 25) into one whitefish scaffold WFS22 that showed inflated coverage estimates across the scaffold, suggesting that both homeologues of the fused PK chromosomes were collapsed in our assembly due to their high sequence similarity. Although their high sequence similarity makes duplicated regions difficult to assemble and subsequently sometimes causes their collapse during assembly, we have assembled a highly continuous reference genome. This is despite a high sequence similarity between large parts of the genome, with at least 80% of the genome being ≥ 90% sequence similar. Our estimates of sequence similarity are comparable to observations in Atlantic salmon where it has been shown that 94% of the chromosome sequence is duplicated, with 26% of the genome having a duplicate region with sequence similarity > 90% (Lien et al., 2016). Future work should aim to investigate the partially collapsed scaffolds WFS04, WFS07, WFS17, and WFS37 and collapsed regions which span the length of scaffolds WFS22, WFS28, WFS32, WFS35, and WFS38, which we identified (Figures 4 and 7; Table S3), to determine the evolutionary history of these duplicates and the process by which the nonduplicated regions of the whitefish genome may have rediploidized independently, or not, compared with other salmonid species. Specifically, it should be determined whether the lack of identified homeologues for WFS22, WFS28, WFS32, WFS35, and WFS38 is an artefact of our assembly (as indicated by their increased coverage [Figure 4]; for example for WFS28, WFS32, and WFS38), the result of genomic rearrangements resulting in coregonid‐specific arrangement (such as WFS39), or a combination of the two (such as WFS22).

In addition to facilitating the investigation of salmonid genome evolution, the highly contiguous whitefish assembly presented here will also support future genomic studies within the subfamily Coregoninae. Coregoninae are distributed across the northern hemisphere (North America and Eurasia), widely fished and of economic importance, and exhibit an extraordinary ecological diversity. Studying whitefish diversification is of fundamental scientific interest to understand the processes driving and facilitating such diversification and to assist in the conservation of this diverse group. We anticipate that the whitefish genome assembly presented here will aid future investigations into the ecology and evolution of all whitefish. Specifically, it will facilitate investigations into the genetic basis of adaptation across the Alpine whitefish radiation, including determining the level of parallelism across multiple pre‐alpine lake systems at a genome‐wide resolution.

## AUTHOR CONTRIBUTIONS

P.G.D.F. conceived and designed the study. Genome assembly and annotation were carried out by R.D.K. and S.Z., and genome validation by R.D.K. The manuscript was written by R.D.K. and P.G.D.F. Funding was awarded to P.G.D.F. All authors read and revised the manuscript and approved the final version for submission.

## Supporting information

Supplementary Material

## Data Availability

Fastq files for PacBio and Illumina raw reads are deposited at the European Nucleotide Archive (ENA study accession no.: PRJEB33097, sample accession no.: ERS359599). The wtdbg2 reference assembly is available online at the European Nucleotide Archive (accession no.: GCA_902810595.1). The falcon and canu assemblies are available on Dryad: https://doi.org/10.5061/dryad.xd2547ddf. All parameters and scripts for genome analysis are accessible at https://github.com/RishiDeKayne/WhitefishReferenceGenome
