# Supplementary material for: A de novo chromosome‐level genome assembly of Coregonus sp. “Balchen”: One representative of the Swiss Alpine whitefish radiation
Source: Mol Ecol Resour. 2020 May 29;20(4):1093–109. doi: 10.1111/1755-0998.13187 (PMC7497118; doi:10.1111/1755-0998.13187)
Supplement: Supplementary file 1 — Supplementary Material [file MEN-20-1093-s001.pdf]

MOLECULAR ECOLOGY RESOURCES

Supplemental Information for:

A de novo chromosome-level genome assembly of Coregonus sp. "Balchen": one representative of the Swiss Alpine whitefish radiation

Authors: Rishi De-Kayne [1,2], Stefan Zoller [3] and Philine G. D. Feulner [1,2]

Affiliations:
1. Department of Fish Ecology and Evolution, Centre of Ecology, Evolution and Biogeochemistry, EAWAG Swiss Federal Institute of Aquatic Science and Technology, Switzerland
2. Division of Aquatic Ecology and Evolution, Institute of Ecology and Evolution, University of Bern, Switzerland
3. Genetic Diversity Centre (GDC), ETH Zürich, Zürich, Switzerland

Table of Contents:

Figure S1 – Falcon Hi-C Contact Plot	Page 1
Figure S2 – Falcon Illumina Coverage Plot	Page 2
Figure S3 – Falcon Synteny Plot	Page 3
Figure S4 – Canu Hi-C Contact Plot	Page 4
Figure S5 – Canu Illumina Coverage Plot	Page 5
Figure S6 – Canu Synteny Plot	Page 6
Figure S7 – whitefish (wtdbg2 assembly) SyMAP dotplot with Northern Pike genome	Page 7
Table S1 – Assembly summary statistics	Page 8
Table S2 – Assembly summary statistics for the 40 Hi-C scaffolds from each assembly	Page 9
Table S3 – BED file of full/partially collapsed wtdbg2 scaffolds	Page 10
Table S4 – BED file for collapsed regions of each assembly	Page 11
Table S5 – SyMAP output from mapping wtdbg2 scaffold assembly to itself	Page 12-15

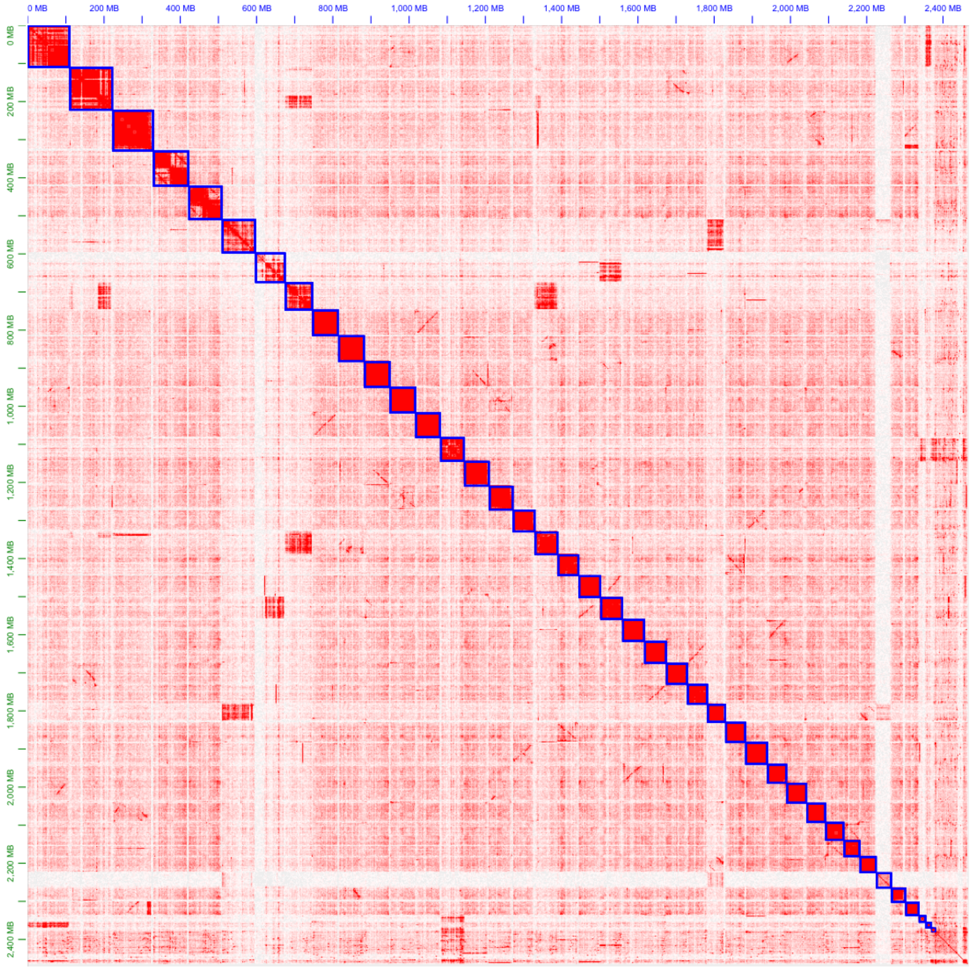

Figure S1. *Coregonus sp. "Balchen"* contig contact map from Hi-C scaffolding of the Falcon assembly. The intensity of red represents the relative contact density between contigs. The highest contact density is found within whitefish scaffolds (FSs), which are outlined in blue.

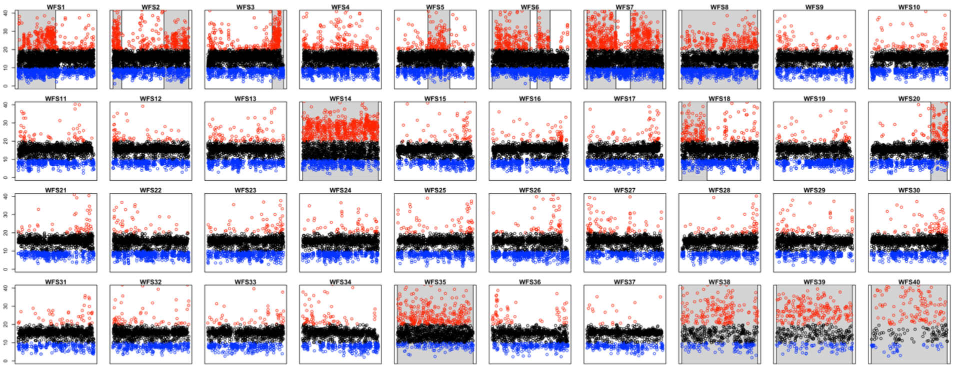

Figure S2. For each of the whitefish scaffolds (FSs) coverage of Illumina data mapped to the Falcon assembly is plotted in 30 Kb windows. Windows with coverage >20, and <10 are coloured in red and blue respectively. Putative collapsed duplicate regions are highlighted in grey.

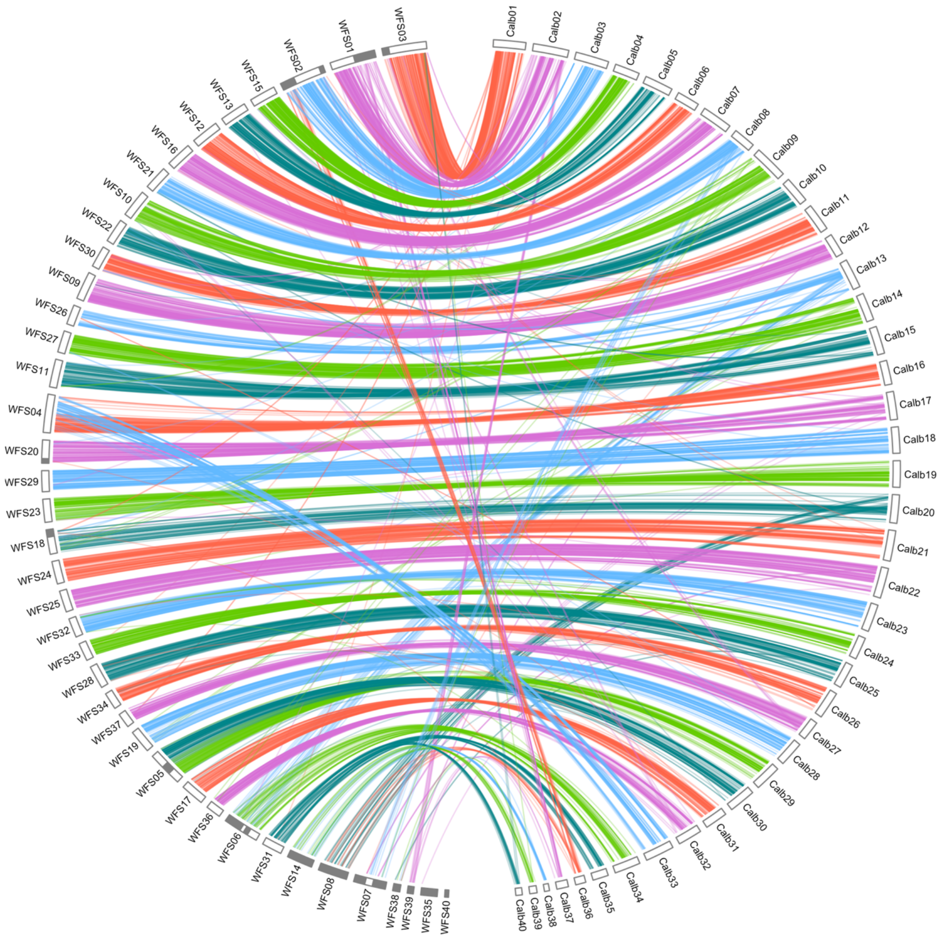

Figure S3. Circos plot comparing the structure of the *C. sp. "Albock"* linkage map (right; De-Kayne & Feulner, 2018) and the 40 whitefish scaffolds (FSs) of the Falcon *C. sp. "Balchen"* assembly (left). Lines indicate mapping locations of RAD loci from the linkage map in the genome assembly. Genome assembly regions which represent collapsed duplicate regions are identified in grey around the left perimeter.

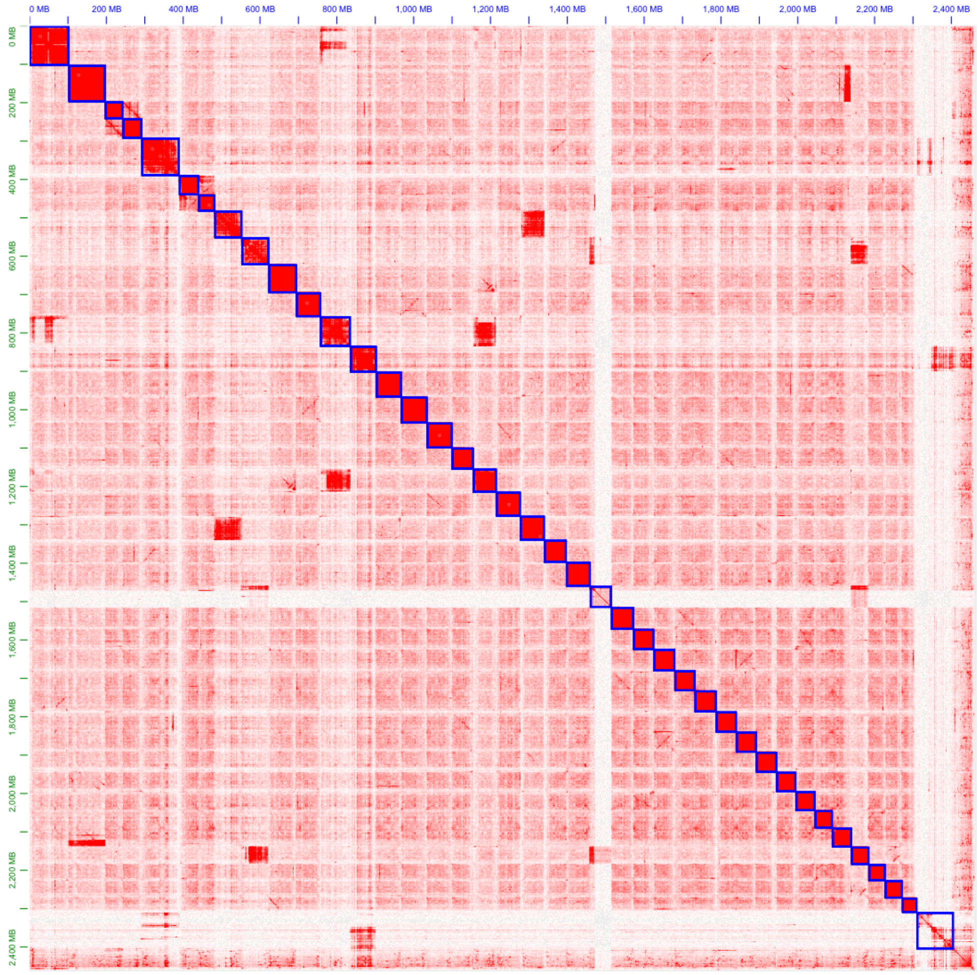

Figure S4. *Coregonus sp. "Balchen"* contig contact map from Hi-C scaffolding of the Canu assembly. The intensity of red represents the relative contact density between contigs. The highest contact density is found within whitefish scaffolds (CSs), which are outlined in blue.

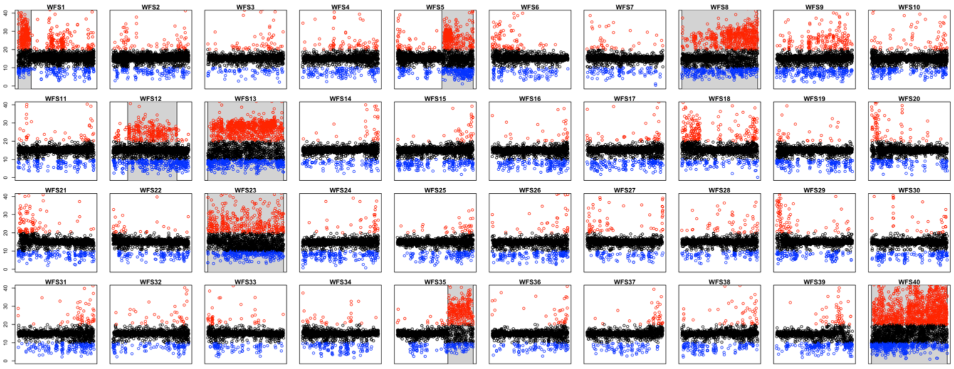

Figure S5. For each of the whitefish scaffolds (CSs) coverage of Illumina data mapped to the Canu assembly is plotted in 30 Kb windows. Windows with coverage >20, and <10 are coloured in red and blue respectively. Putative collapsed duplicate regions are highlighted in grey.

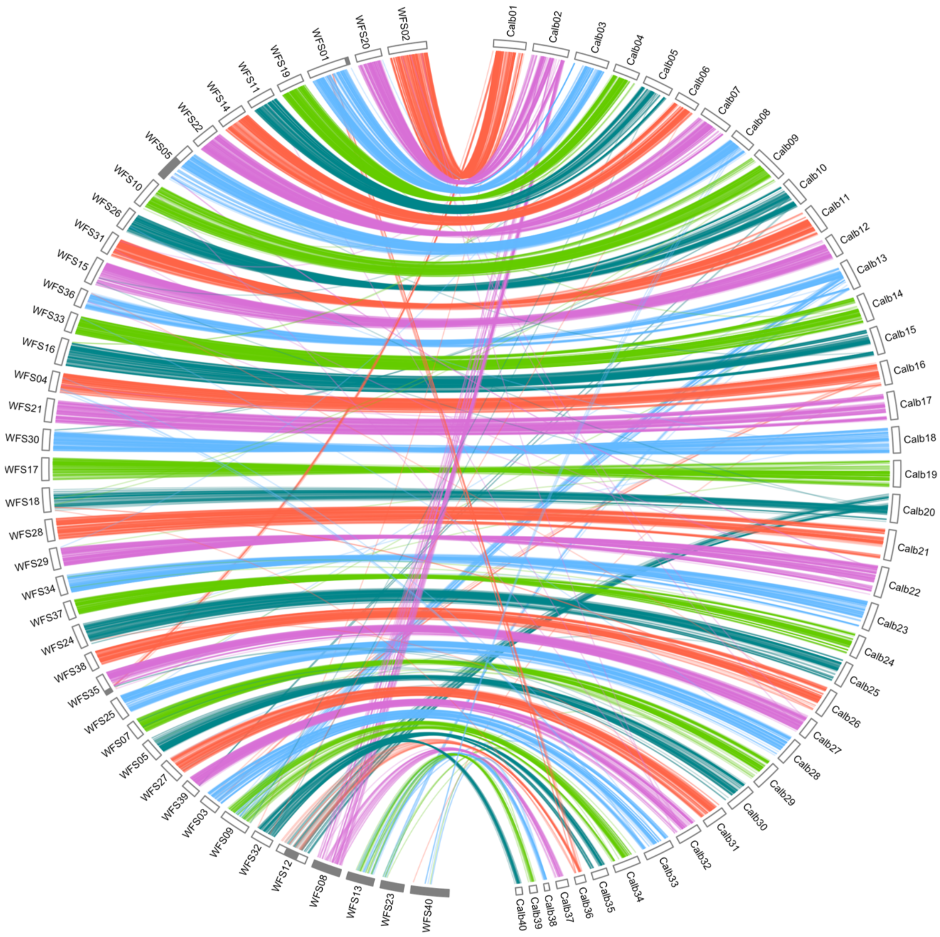

Figure S6. Circos plot comparing the structure of the *C. sp. "Albock"* linkage map (right; De-Kayne & Feulner, 2018) and the 40 whitefish scaffolds (FSs) of the Falcon *C. sp. "Balchen"* assembly (left). Lines indicate mapping locations of RAD loci from the linkage map in the genome assembly. Genome assembly regions which represent collapsed duplicate regions are identified in grey around the left perimeter.

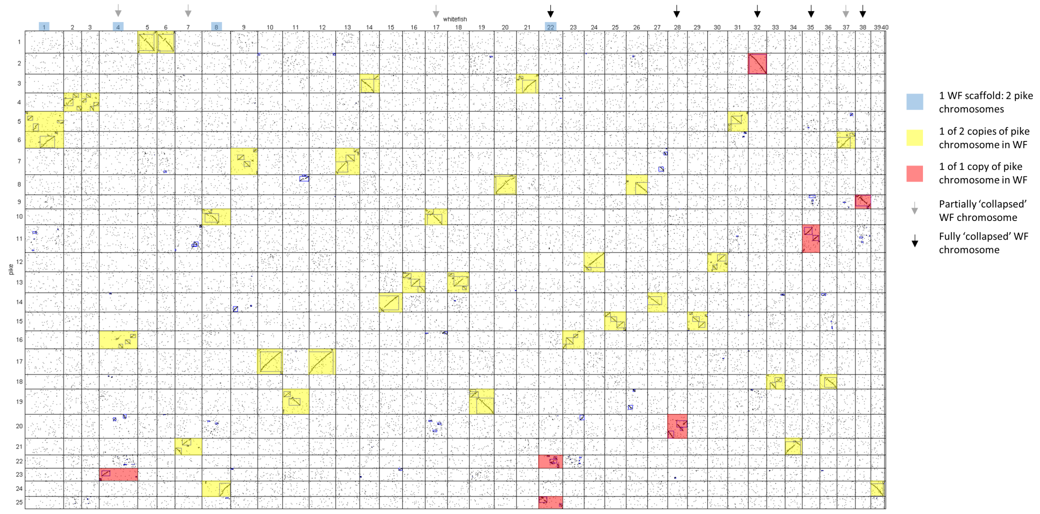

Figure S7. Dot plot showing the results of SyMAP synteny analysis mapping the wtdbg2 WFSs to the Northern Pike genome. Blue highlighting of WFSs indicates that one WFS maps to two pike chromosomes. Yellow highlighting of WFSs indicates that the WFS makes up one of the two whitefish scaffolds that correspond to a single pike chromosome. Red highlighting indicates that a single WFS corresponds to a pike chromosome. Partially collapsed (grey) and fully collapsed (black) WFSs are indicated by an arrow above the WFS name.

Table S1 - summary statistics at each stage of the assembly process

		RAW Falcon (merged p and h)	RAW Canu	RAW wtdbg2	Purged Falcon	Purged Canu	Purged wtdbg2	PG Scaffolded Falcon	PG Scaffolded Canu	PG Scaffolded wtdbg2
file		cns_PandH_ctg.renamed_polished.fasta	canu1.6.v2.pilon.iter1.fasta	wtdbg2.arrow.iter1.fasta	Falcon.purged.ordered.fasta	Canu.purged.ordered.fasta	wtdbg2.purged.ordered.fasta			
stats	n	60605	52023	28224	19412	22627	16440	3745	3553	7855
	n:500	60605	52023	28224	19412	22627	16440	3745	3553	7855
	n:N50	5828	4228	1276	1889	1986	1074	16	17	18
	min	569	1200	1722	569	1392	2330	569	999	999
	N80	38612	36907	82666	84828	69497	131893	51330000	50370000	43650000
	N50	136418	130955	424474	281384	258083	491356	62840000	60610000	51930000
	N20	567511	669827	1285210	897953	876095	1379796	87800000	77010000	63860000
	max	6516619	5278180	5201837	6516619	5278180	5201837	111200000	104000000	93420000
	sum	4.11E+09	3.28E+09	2.38E+09	2.47E+09	2.46E+09	2.20E+09	2.47E+09	2.46E+09	2.20E+09
busco	Complete	89.5	88.7	93.4	90.8	93.1	93.1	91.8	93.7	93.3
	Single	26.9	39.4	55.8	43.6	49.1	55.6	59.1	58.3	55.7
	Duplicated	62.6	49.3	37.6	47.2	44	37.5	32.7	35.4	37.6
	Fragmented	2.8	5	2.2	2.4	2.2	2.2	1.9	1.9	2.1
	Missing	7.7	6.3	4.4	6.8	4.7	4.7	6.3	4.4	4.6
	number	4584	4584	4584	4584	4584	4584	4584	4584	4584
repeats	% masked				58.64	58.25	52.72	52.01	51.78	52.08
	GC content				43.68	43.74	43.88	43.68	43.74	43.88
	SINEs				1.33	1.33	0.32	0.25	0.28	0.27
	LINEs				11.41	11.54	11.05	10.61	10.78	11.02
	LTR elements				5.35	5.48	4.7	4.97	5.11	4.67
	DNA elements				23.13	22.33	24.54	24.65	23.79	24.41
	Unclassified				14.22	14.1	8.5	8.48	8.61	8.13
	Total interspersed repeats				55.45	54.79	49.11	48.95	48.57	48.5
	Small RNA				0.03	0.05	0.04	0.04	0.06	0.04
	Satellites				0.46	0.45	0.26	0.25	0.25	0.23
	Simple repeats				2.59	2.86	3.02	2.6	2.77	3
	Low complexity				0.37	0.36	0.51	0.37	0.33	0.52
Purging parameters	low				1	2	10			
	mid				55	55	55			
	high				120	125	120			
Purging parameters					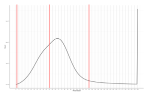	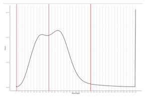	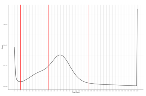			
Description		This assembly is made by concatenating the primary and haplotig assemblies provided by DNAnexsus polished with arrow then pilon	This assembly is the Canu output v2 from euler polished with pilon x1	This assembly is the polished output from wtdbg2 v2 -arrow and pilon polished	This is the RAW Falcon (merged p and h) assembly after purge_haplotigs	This is the RAW Canu assembly after purge_haplotigs	Ths is the RAW wtdbg assembly after purge_haplotigs	This assembly is made from Falcon.purged.ordered.fasta and scaffolded by Phase Genomics into 40 chromosome-scale scaffolds	This assembly is made from Canu.purged.ordered.fasta and scaffolded by Phase Genomics into 40 chromosome-scale scaffolds	This assembly is made from wtdbg2.purged.ordered.fasta and scaffolded by Phase Genomics into 40 chromosome-scale scaffolds

Table S2 - assembly statistics for the 40 chromosome-scale scaffolds and statistics from assembly validation using Illumina data and linkage map

			Falcon	Canu	wtdbg2
**Statistics for 40 chromosome-scale scaffolds**	**BUSCOs**	Complete	91.5	93.7	93
		Single	59.6	56.3	55.7
		Duplicated	31.9	37.4	37.3
		Fragmented	1.7	1.7	1.8
		Missing	6.8	4.6	5.2
		number	4584	4584	4584
	**Assembly statistics**	n	40	40	40
		n:500	40	40	40
		n:N50	15	16	17
		min	9.36E+06	2.45E+07	1.09E+06
		N80	5.25E+07	5.22E+07	4.46E+07
		N50	6.30E+07	6.13E+07	5.20E+07
		N20	8.78E+07	9.03E+07	6.39E+07
		max	1.11E+08	1.04E+08	9.34E+07
		sum	2.38E+09	2.41E+09	2.07E+09
**Genome validation**	**Illumina coverage**	total Illumina reads	578426578	578426578	578426578
		number mapped	572316621	579664797	584498965
		number mapped > 30 mapq	347556473	402701783	418284790
		% > 30 mapq	60.73	69.47	71.56
		mate mapped to different chro	11671519	8345236	14053664
		% mate mapped to different c	2.04	1.44	2.40
		mean coverage	14.99976	15.89349	17.30452
		total windows	79366	80237	68916
		# windows <= 10	18141	7017	1916
		% <= 10	22.86	8.75	2.78
		# windows 11-19	54987	66188	57172
		% 11-19	69.28	82.49	82.96
		# windows >= 20	6238	7032	9828
		% >= 20	7.86	8.76	14.26
		# windows < 60x coverage	79046	79890	68571
		# over coverage windows	320	347	345
		% windows extreme cov	0.40	0.43	0.50
		# windows < 30x coverage	77669	78196	66561
		# over coverage windows	1697	2041	2355
		% windows extreme cov	2.14	2.54	3.42
	**Linkage map**	number of markers > 30 mapq	3713	4538	4813
		number of markers on chromo	3648	4494	4744
		% of 5395 markers on chromos	67.62	83.30	87.93
		number of markers = 60 mapq	3168	3931	4197
		percentage = 60	86.84	87.47	88.47

Table S3 - BED file for partial and fully collapsed wtdbg2 scaffolds (WFSs)

WFS	start	end	description
4	0	31500000	partially collapsed scaffold
7	39000000	65391737	partially collapsed scaffold
17	41500000	54216998	partially collapsed scaffold
37	32500000	43663377	partially collapsed scaffold
22	0	56862223	fully collapsed scaffold
28	0	46671285	fully collapsed scaffold
32	0	44616205	fully collapsed scaffold
35	0	42609905	fully collapsed scaffold
38	0	36774138	fully collapsed scaffold

Table S4 - BED files for the partial and fully collapsed scaffolds for the wtdbg2, Canu, and Falcon assemblies

wtdbg2 - WFS	start	end	length	Canu - CS	start	end	length	Falcon - FS	start	end	length
4	0	31500000	31500000	1	0	17900000	17900000	1	0	55000000	55000000
7	39000000	65391737	26391737	5	58000000	98850821	40850821	2	0	13000000	13000000
17	41500000	54216998	12716998	12	15000000	65000000	50000000	2	75000000	111403654	36403654
37	32500000	43663377	11163377	35	33000000	49519047	16519047	3	91000000	108247109	17247109
22	0	56862223	56862223	8	0	70670027	70670027	5	35000000	60000000	25000000
28	0	46671285	46671285	13	0	66680231	66680231	6	0	44000000	44000000
32	0	44616205	44616205	23	0	56808971	56808971	6	50000000	65000000	15000000
35	0	42609905	42609905	40	0	93690684	93690684	7	0	30000000	30000000
38	0	36774138	36774138					7	45000000	78496923	33496923
								8	0	71144181	71144181
								14	0	62908580	62908580
								18	0	20000000	20000000
								20	44000000	56202205	12202205
								35	0	39938056	39938056
								38	0	17454843	17454843
								39	0	14814143	14814143
								40	0	9379409	9379409
		total collapsed	309305868			total collapsed	413119781			total collapsed	516989103
		proportion	14.0626335			proportion	16.7725828			proportion	20.9555603

Table S5 - SyMAP output for mapping of each wtdbg2 scaffold (WFS) against every other WFS (raw data used to plot Figure 7)

Species1	Species2	Chr1	Chr2	BlkNum	Start1	End1	Start2	End2	#Hits	Genes1	%Genes1	Genes2	%Genes2	PearsonR
whitefish	whitefish	1	31	1	6522252	29578054	1464877	22277307	161	0	0	0	0	-0.969364
whitefish	whitefish	1	31	2	128552	6295004	44478765	47995089	23	0	0	0	0	-0.957101
whitefish	whitefish	1	31	3	68076591	69001314	35621901	36246350	16	0	0	0	0	0.9574
whitefish	whitefish	1	31	4	29741600	32248056	5363250	8244269	28	0	0	0	0	0.998757
whitefish	whitefish	1	31	5	84053008	85184111	32391907	33967409	8	0	0	0	0	0.981619
whitefish	whitefish	1	31	6	78218434	91829077	22487546	32813664	110	0	0	0	0	0.804091
whitefish	whitefish	1	31	7	69059781	79323833	36332799	44424120	58	0	0	0	0	0.990293
whitefish	whitefish	1	37	1	33788451	67518464	3520370	31770713	302	0	0	0	0	0.991347
whitefish	whitefish	2	3	1	12741283	43024459	357982	28905327	405	0	0	0	0	0.982829
whitefish	whitefish	2	3	2	11677522	12831119	30089730	31420742	28	0	0	0	0	-0.965301
whitefish	whitefish	2	3	3	7289185	11071158	36926305	42158190	76	0	0	0	0	-0.934762
whitefish	whitefish	2	3	4	1652625	6319575	31912860	35756303	52	0	0	0	0	0.931324
whitefish	whitefish	3	2	1	357982	28905327	12741283	43024459	405	0	0	0	0	0.982829
whitefish	whitefish	3	2	2	30089730	31420742	11677522	12831119	28	0	0	0	0	-0.965301
whitefish	whitefish	3	2	3	36926305	42158190	7289185	11071158	76	0	0	0	0	-0.934762
whitefish	whitefish	3	2	4	31912860	35756303	1652625	6319575	52	0	0	0	0	0.931324
whitefish	whitefish	4	14	1	87345185	90387377	697444	4016606	59	0	0	0	0	-0.897659
whitefish	whitefish	4	23	1	45220803	87193811	2533344	39637472	310	0	0	0	0	0.959643
whitefish	whitefish	4	23	2	31738231	44949152	11202243	46596690	163	0	0	0	0	-0.829482
whitefish	whitefish	4	23	3	58764583	62558050	47075592	51797507	32	0	0	0	0	-0.931978
whitefish	whitefish	5	6	1	62806	44699089	542401	43479891	695	0	0	0	0	0.998576
whitefish	whitefish	6	5	1	542401	43479891	62806	44699089	695	0	0	0	0	0.998576
whitefish	whitefish	7	34	1	1645699	9924584	6356259	14257332	48	0	0	0	0	-0.992108
whitefish	whitefish	7	34	2	31553219	38202933	30626247	39596289	26	0	0	0	0	0.841465
whitefish	whitefish	7	34	3	16731933	33723790	18432223	35969855	228	0	0	0	0	-0.994862
whitefish	whitefish	7	34	4	10891858	15957935	367570	5501408	43	0	0	0	0	-0.998169
whitefish	whitefish	7	34	5	56570604	56989624	14513892	14926933	15	0	0	0	0	0.996667
whitefish	whitefish	7	35	1	595244	1576585	32207	1308784	14	0	0	0	0	-0.987397
whitefish	whitefish	8	17	1	11790513	40755800	5924874	39526914	400	0	0	0	0	0.948384
whitefish	whitefish	8	17	2	243516	10742105	266972	18102533	48	0	0	0	0	0.850324
whitefish	whitefish	8	17	3	10888226	11480055	15200700	15764432	9	0	0	0	0	-0.992559
whitefish	whitefish	9	13	1	17263429	47326144	14913600	42075749	232	0	0	0	0	-0.987314
whitefish	whitefish	9	13	2	51065298	63762662	42391536	56202959	86	0	0	0	0	0.980967
whitefish	whitefish	9	15	1	43990405	50935842	14828060	20297719	49	0	0	0	0	-0.9923
whitefish	whitefish	9	15	2	8639274	15472022	210032	7807370	67	0	0	0	0	-0.981179
whitefish	whitefish	9	15	3	15590166	16809204	13167929	14620719	18	0	0	0	0	0.965025
whitefish	whitefish	9	15	4	2961231	6544542	49824580	55625040	35	0	0	0	0	-0.806418
whitefish	whitefish	10	12	1	6975565	56689206	4384342	57188085	650	0	0	0	0	0.997276

whitefish	whitefish	10	12	2	383411	9900559	51580030	62542285	44	0	0	0	0	-0.834843
whitefish	whitefish	10	12	3	48432928	60271634	169784	21687881	20	0	0	0	0	-0.943525
whitefish	whitefish	11	19	1	582569	41618642	4816258	41914059	379	0	0	0	0	0.990678
whitefish	whitefish	11	20	1	41958972	58559889	34024345	53626288	181	0	0	0	0	0.921818
whitefish	whitefish	11	20	2	40749182	62844935	228060	19194662	29	0	0	0	0	-0.940342
whitefish	whitefish	12	10	1	4384342	57188085	6975565	56689206	650	0	0	0	0	0.997276
whitefish	whitefish	12	10	2	51580030	62542285	383411	9900559	44	0	0	0	0	-0.834843
whitefish	whitefish	12	10	3	169784	21687881	48432928	60271634	20	0	0	0	0	-0.943525
whitefish	whitefish	13	9	1	14913600	42075749	17263429	47326144	232	0	0	0	0	-0.987314
whitefish	whitefish	13	9	2	42391536	56202959	51065298	63762662	86	0	0	0	0	0.980967
whitefish	whitefish	13	27	1	943775	14781495	27648517	40386364	161	0	0	0	0	-0.97225
whitefish	whitefish	14	4	1	697444	4016606	87345185	90387377	59	0	0	0	0	-0.897659
whitefish	whitefish	14	21	1	4717464	43081967	6476166	52428468	441	0	0	0	0	-0.993324
whitefish	whitefish	14	21	2	42320226	46619162	47202432	51822135	16	0	0	0	0	0.880332
whitefish	whitefish	15	9	1	14828060	20297719	43990405	50935842	49	0	0	0	0	-0.9923
whitefish	whitefish	15	9	2	210032	7807370	8639274	15472022	67	0	0	0	0	-0.981179
whitefish	whitefish	15	9	3	13167929	14620719	15590166	16809204	18	0	0	0	0	0.965025
whitefish	whitefish	15	9	4	49824580	55625040	2961231	6544542	35	0	0	0	0	-0.806418
whitefish	whitefish	15	27	1	18334123	47003557	87375	30273258	267	0	0	0	0	-0.971657
whitefish	whitefish	16	18	1	8976484	48538158	2421000	44404315	564	0	0	0	0	0.997382
whitefish	whitefish	16	18	2	635991	9012562	45051285	51579834	52	0	0	0	0	-0.876748
whitefish	whitefish	17	8	1	5924874	39526914	11790513	40755800	400	0	0	0	0	0.948384
whitefish	whitefish	17	8	2	266972	18102533	243516	10742105	48	0	0	0	0	0.850324
whitefish	whitefish	17	8	3	15200700	15764432	10888226	11480055	9	0	0	0	0	-0.992559
whitefish	whitefish	18	16	1	2421000	44404315	8976484	48538158	564	0	0	0	0	0.997382
whitefish	whitefish	18	16	2	45051285	51579834	635991	9012562	52	0	0	0	0	-0.876748
whitefish	whitefish	19	11	1	4816258	41914059	582569	41618642	379	0	0	0	0	0.990678
whitefish	whitefish	19	26	1	35859157	49948638	409683	15430177	85	0	0	0	0	0.968655
whitefish	whitefish	19	26	2	89727	4497497	16238015	21548041	31	0	0	0	0	0.958293
whitefish	whitefish	20	11	1	34024345	53626288	41958972	58559889	181	0	0	0	0	0.921818
whitefish	whitefish	20	11	2	228060	19194662	40749182	62844935	29	0	0	0	0	-0.940342
whitefish	whitefish	20	26	1	6156553	33951348	91192	49388898	351	0	0	0	0	-0.820859
whitefish	whitefish	21	14	1	6476166	52428468	4717464	43081967	441	0	0	0	0	-0.993324
whitefish	whitefish	21	14	2	47202432	51822135	42320226	46619162	16	0	0	0	0	0.880332
whitefish	whitefish	23	4	1	2533344	39637472	45220803	87193811	310	0	0	0	0	0.959643
whitefish	whitefish	23	4	2	11202243	46596690	31738231	44949152	163	0	0	0	0	-0.829482
whitefish	whitefish	23	4	3	47075592	51797507	58764583	62558050	32	0	0	0	0	-0.931978
whitefish	whitefish	24	30	1	37669822	50310481	171974	14062732	57	0	0	0	0	-0.924892
whitefish	whitefish	24	30	2	2286436	43205815	7671836	44680332	473	0	0	0	0	0.876873
whitefish	whitefish	24	30	3	13626981	18645123	41786865	47675552	40	0	0	0	0	-0.941397

whitefish	whitefish	25	29	1	5811697	43229745	5254275	41685543	532	0	0	0	0	0.996478
whitefish	whitefish	25	29	2	399291	18852816	33899924	48133499	55	0	0	0	0	-0.933
whitefish	whitefish	26	19	1	409683	15430177	35859157	49948638	85	0	0	0	0	0.968655
whitefish	whitefish	26	19	2	16238015	21548041	89727	4497497	31	0	0	0	0	0.958293
whitefish	whitefish	26	20	1	91192	49388898	6156553	33951348	351	0	0	0	0	-0.820859
whitefish	whitefish	27	13	1	27648517	40386364	943775	14781495	161	0	0	0	0	-0.97225
whitefish	whitefish	27	15	1	87375	30273258	18334123	47003557	267	0	0	0	0	-0.971657
whitefish	whitefish	29	25	1	5254275	41685543	5811697	43229745	532	0	0	0	0	0.996478
whitefish	whitefish	29	25	2	33899924	48133499	399291	18852816	55	0	0	0	0	-0.933
whitefish	whitefish	30	24	1	171974	14062732	37669822	50310481	57	0	0	0	0	-0.924892
whitefish	whitefish	30	24	2	7671836	44680332	2286436	43205815	473	0	0	0	0	0.876873
whitefish	whitefish	30	24	3	41786865	47675552	13626981	18645123	40	0	0	0	0	-0.941397
whitefish	whitefish	31	1	1	1464877	22277307	6522252	29578054	161	0	0	0	0	-0.969364
whitefish	whitefish	31	1	2	44478765	47995089	128552	6295004	23	0	0	0	0	-0.957101
whitefish	whitefish	31	1	3	35621901	36246350	68076591	69001314	16	0	0	0	0	0.9574
whitefish	whitefish	31	1	4	5363250	8244269	29741600	32248056	28	0	0	0	0	0.998757
whitefish	whitefish	31	1	5	32391907	33967409	84053008	85184111	8	0	0	0	0	0.981619
whitefish	whitefish	31	1	6	22487546	32813664	78218434	91829077	110	0	0	0	0	0.804091
whitefish	whitefish	31	1	7	36332799	44424120	69059781	79323833	58	0	0	0	0	0.990293
whitefish	whitefish	33	36	1	9265727	43837681	1794668	41769915	401	0	0	0	0	-0.87024
whitefish	whitefish	33	36	2	785383	10537802	571666	15196051	64	0	0	0	0	0.824279
whitefish	whitefish	34	7	1	6356259	14257332	1645699	9924584	48	0	0	0	0	-0.992108
whitefish	whitefish	34	7	2	30626247	39596289	31553219	38202933	26	0	0	0	0	0.841465
whitefish	whitefish	34	7	3	18432223	35969855	16731933	33723790	228	0	0	0	0	-0.994862
whitefish	whitefish	34	7	4	367570	5501408	10891858	15957935	43	0	0	0	0	-0.998169
whitefish	whitefish	34	7	5	14513892	14926933	56570604	56989624	15	0	0	0	0	0.996667
whitefish	whitefish	34	35	1	8221338	24557252	6091435	32641164	14	0	0	0	0	0.959164
whitefish	whitefish	35	7	1	32207	1308784	595244	1576585	14	0	0	0	0	-0.987397
whitefish	whitefish	35	34	1	6091435	32641164	8221338	24557252	14	0	0	0	0	0.959164
whitefish	whitefish	36	33	1	1794668	41769915	9265727	43837681	401	0	0	0	0	-0.87024
whitefish	whitefish	36	33	2	571666	15196051	785383	10537802	64	0	0	0	0	0.824279
whitefish	whitefish	37	1	1	3520370	31770713	33788451	67518464	302	0	0	0	0	0.991347
